# Prevalence, risk and resilience factors of mental health conditions among female sex workers: a systematic review and meta-analysis

**DOI:** 10.3389/fpubh.2024.1455999

**Published:** 2025-01-13

**Authors:** Olivia Kalinowski, Anastasiia Lotysh, Gizem Kaya, Franziska Kroehn-Liedtke, Lena Karoline Zerbe, Hristiana Mihaylova, Krisztina Sipos, Wulf Rössler, Meryam Schouler-Ocak

**Affiliations:** Department of Psychiatry and Neurosciences, Psychiatric University Clinic of Charité at St. Hedwig Hospital, Berlin, Germany

**Keywords:** mental health, sex work, systematic review, meta-analysis, risk factors

## Abstract

**Introduction:**

Female sex workers are a vulnerable hard-to-reach group. Research in this field is scarce due to several issues, such as methodological difficulties or societal stigmatization. Most of the available literature focuses on sexually transmittable diseases. This review and meta-analysis aim to compile literature on the mental health of female sex workers. We investigated the prevalence of as well as risk factors for mental disease among female sex workers globally.

**Methods:**

Utilizing Preferred Reporting Items for Systematic Reviews and Meta-Analyses (PRISMA) guidelines, we conducted a comprehensive search across several databases, ultimately analyzing data from 80 studies comprising 24,675 individuals in total.

**Results:**

Most of the studies stemmed from the United States (*n* = 24), followed by China (*n* = 12), India (*n* = 7) and Kenya (*n* = 5). Four studies were conducted in South Africa and three in Mexico. Two studies originated from Australia, Cambodia, Thailand, the Netherlands, and Uganda. Single studies were identified from Scotland, Switzerland, Israel, Portugal, Mongolia, Malawi, Cameroon, Ukraine, Togo, Lebanon, the Dominican Republic, Tanzania, Puerto Rico, Ethiopia, and Moldova. The review highlights significant heterogeneity in the prevalence of mental health issues such as anxiety, depression, suicidality, post-traumatic stress disorder (PTSD), substance use and dependence, investigating the influence of socio-economic, legal, and individual factors on these outcomes. The meta-analysis reveals that while factors like legal status of sex work and economic conditions did not show any impact, specific demographic characteristics, notably female sex workers living with human immunodeficiency virus (HIV), migrant female sex workers, or female sex workers engaged in substance use, exhibit notably higher mental health challenges.

**Discussion:**

These findings suggest the critical need for targeted mental health interventions and policy reforms that consider the complex interplay of various factors affecting sex workers. Future research should focus on under-researched regions and subgroups within this population to enhance understanding and support the development of comprehensive health services.

**Systematic review registration:**

PROSPERO, CRD42022312737, available from: https://www.crd.york.ac.uk/prospero/display_record.php?ID=CRD42022312737.

## 1 Introduction

Sex work (SW) as a societal aspect is highly controversial (1). Estimated numbers suggest that globally, 40–42 million people are involved in SW with over 80% of them being women (2). While a proportion of female sex workers consider it a profession, an act of emancipation and self-determination, another proportion works under coercion (total proportions unknown). Regardless of their working conditions, sex workers often face societal stigma and marginalization (3). On the other hand, there are dissenting opinions regarding SW as an act of mostly male violence against women and as a harmful and traumatic experience (4, 5). Additionally, SW is linked to criminal activities such as human trafficking for sexual exploitation, drug abuse, and procurement in certain cases (6, 7). Previous research exploring the physical and mental health challenges encountered by sex workers, predominantly focused on particular concerns like human immunodeficiency virus (HIV) and sexually transmitted infections (STIs) (8) but also discussed various factors related to mental health such as childhood maltreatment and traumatization (with prevalences ranging up to 56.8%) as well as experiencing violence in this profession (with prevalences ranging up to 69.6% for experiencing physical attacks) (9, 10). Studies exploring the connection between prostitution, a history of childhood sexual abuse, and symptoms of post-traumatic stress disorder (PTSD) and other stress-related disorders reveal higher prevalences in sex workers compared to control groups (e.g., 26.2% among sex workers vs. 10.3% among the control group for PTSD) (10, 11). Additionally, the high level of violence experienced by sex workers has been linked to mental health issues and was addressed in previous studies, showing, e.g., that individuals who experienced any or combined forms of violence (physical, sexual, verbal) in the workplace are 1.76 times more likely to have depression compared to those who did not experience such violence (12). Research indicates that sex workers encounter violence more frequently than comparison groups before entering SW. Therefore, it is likely that sex workers, like other marginalized groups, exhibit poorer mental health compared to the general population (13–15). A study in Switzerland revealed high 1-year prevalences of mental disorders among female sex workers, associated with experiences of violence and the perceived burden of SW: annual prevalences of affective disorders were found to be six times higher than in the general population, similar to anxiety disorders (16). The frequency of mental disorders is linked to specific working conditions (e.g., in escort services, brothels or street prostitution). According to Rossler et al. (16), for example, 12% of women working in a street setting were found to display current PTSD symptoms, while none of the women in a studio setting were affected by PTSD (14, 16). Migration background of sex workers and therefore the right of residence also plays a significant role in shaping these conditions. In Zurich, for example, a prevalence of 47% for depression was found among migrant sex workers while only 10% of Swiss women suffered from depression (16). The findings underscore that the psychological challenges faced by sex workers are partially tied to job-specific risks, including exposure to violence and the lack of adequate social support (8).

Comprehensive data on sex workers’ mental health, the burdens they face, and, in this context, the role of violence are limited. To address this gap and compile stress and resilience factors contributing to the mental health of female sex workers were the aims of this systematic review and meta-analysis. The review seeks to quantify the prevalence of common mental health conditions such as anxiety disorders, depression, PTSD, suicidality, and substance use among female sex workers as well as provide an overview of the effect sizes of associated risk and resilience factors. Thereby, we aim to highlight important public health issues and outline current research gaps. By aggregating data from various studies for the prevalences of several mental diseases, this meta-analysis provides a more robust estimate of the mental health burden in this population. Additionally, we conduct a subgroup analysis based on the legal situation (legal, partially legal and illegal), as well as on the national economic situation based on the gross domestic product (GDP). Specific subgroups of female sex workers, namely those living with HIV, drug-using sex workers, migrant sex workers, and survivors of sex trafficking, will be tested as mediators of prevalences. To provide a comprehensive analysis, this systematic review and meta-analysis include studies from a wide range of geographic regions without any geographical restrictions.

## 2 Methods

This review was preregistered on Prospero (ID: CRD42022312737) and follows the Preferred Reporting Items for Systematic Reviews and Meta-Analyses (PRISMA) guidelines. This systematic review and meta-analysis were conducted as part of a cross-sectional prevalence study regarding the mental health of sex workers in Berlin. The project was funded by the German Research Association (DFG); the grant ID is GZ: SCHO 772/4–1 and GZ: RO 948/7–1.

### 2.1 Inclusion and exclusion criteria

The studies included in the review had to meet the following criteria: (a) be quantitative studies with samples made up of sex workers and/or other population groups for comparative purposes; (b) be written in English or German; (c) be published after 2002 in peer-reviewed journals; (d) include a sample composed of adult female sex workers (18–65 years); and (e) investigate the relationship between sex work and mental health issues such as depression, suicidality, anxiety, substance abuse, psychotic disorders, post-traumatic stress disorders, and others risk and resilience factors. Qualitative studies were excluded, as well as studies not providing data on mental health, studies focused on sexually transmittable diseases (STDs), and studies focused on men, trans-gender sex workers or minors as they probably have different biographic experiences, issues with self-esteem, and stigma. The outcomes of interest were prevalences of mental disease and risk and resilience factors for mental disorders among female sex workers. Experience of violence and childhood trauma were considered as risk factors for mental disorders. The prevalence of violence and childhood trauma as primary outcomes was not assessed in this study, due to the amount of available literature.

### 2.2 Search strategy

Rayyan 20 (17) was used to screen the literature. The search was conducted in Medline via Web of Science, Cochrane, PubPsych, Embase Classic + Embase, and APA PsycINFO via EBSCOhost. A systematic search was performed including Medical Subject Headings (MeSH) terms as well as specific keywords related to the topic of research. The MeSH terms used in our search strategy are provided in the Supplementary Table 1, which also includes the search terms for all databases where the search was conducted. The Peer Review of Electronic Search Strategy (PRESS) checklist (18) was used to control the correct conduction of literature research. All searches were performed in English or German. The search was restricted to articles published between 2002 and 2023 to ensure that the analysis reflects the most relevant and up-to-date research on the mental health of female sex workers. Following the global recognition of the HIV epidemic and its impact on sex workers in the early 2000s, there was a surge in research focusing not only on physical health but also on the mental health outcomes of female sex workers. This period thus represents a turning point where mental health became a more central component of research related to sex workers. We initially set out to investigate studies from the past 20 years, starting our review preparations in 2022. The first literature search was conducted in October 2023. In January 2024, we updated the literature search to include studies from 2023 to ensure that our analysis reflects the most current research and developments in the field. A manual search of the reference lists of included studies was conducted to identify additional relevant publications. We chose this approach because the research of interest is highly resource-intensive (e.g., time-consuming regarding participant recruitment or financially in terms of participation incentives and staff salaries), and we determined that scientific journals would likely provide a more comprehensive and reliable source of relevant literature compared to grey literature, such as policy papers or blog posts. The search was conducted in multiple steps and performed by three professional independent staff members, of which one had an academic background in psychology, one in psychology and public health, and one in medicine. First, a title screening was conducted. Secondly, an abstract screening was performed and followed by a full-text screening. Each screening was conducted by the three staff members and disagreements were resolved in each case by checking the full text for the inclusion criteria (see section 2.1).

### 2.3 Data extraction and quality of studies

For each study, the staff members who conducted the screening extracted the following data into an Excel^®^ spreadsheet: ID, topic, first author, year of publication, title, journal, volume, place, country, study design, setting, population, sample size, inclusion/exclusion criteria, recruitment, interventions, start-end date, main outcomes/assessment points, study instruments and quality of study instruments, comparison/control group, main findings, size of effect. For a comprehensive overview, we report the following data from the studies that met the inclusion criteria in this review and meta-analysis: Author, year, the legal status of sex work, sample size and population, study design, recruitment strategy, as well as measurements of the outcome variables and results. The methodological quality of all included studies was assessed by the same three staff members independently and systematically, using the Newcastle Ottawa Quality Assessment Scale (NOS). Only studies using validated standardized instruments and scoring at least 3 points were included.

### 2.4 Meta-analysis

Meta-analysis was conducted using the “metafor” package in R (19, 20). We calculated the logit-transformed prevalence from the reported prevalence rates to stabilize the variances and used the reported confidence intervals to calculate the standard errors of the logit-transformed prevalence. If the 95% confidence intervals (CI) were not reported, we calculated the 95% CI from the sample size and the given prevalence using the Wilson score method, as it provides more accurate confidence intervals particularly when dealing with proportions close to the boundaries (0 or 1) or when sample sizes are relatively small (21). We adopted a random-effects model due to the anticipated heterogeneity among studies, quantified using the I^2^ statistic, which describes the percentage of total variation across studies due to heterogeneity rather than chance. Subgroup analyses were performed to explore the potential effects of poverty (assessed through the country’s GDP per capita), legal status of sex work, specific sub-populations, and assessment method [clinical interview versus other (self-) assessment tool] on prevalence. We hypothesized that the economic status of a country, as indicated by GDP per capita, influences access to essential resources such as food, water, and healthcare, which in turn may mediate the prevalence of mental health conditions. While GDP per capita is not a direct measure of resource access, it is widely used as a proxy for a country’s overall economic capacity to provide these resources. To capture these effects, we analyzed the data using meta-regression to examine the relationship between GDP per capita, treated as a continuous variable, and the effect sizes of mental health outcomes across studies. Legal status was considered as a categorical variable, reflecting the legal framework governing sex work in the study location: “legal,” “partially legal,” and “illegal.” “Partially legal” comprises legislature where, e.g., sex work is legal, but running sex work establishments, such as brothels, is illegal. Population subgroups included, e.g., sex workers living with HIV, drug-using sex workers, or survivors of human trafficking. To account for the risk of Type I error due to multiple comparisons, we applied a Bonferroni correction (22) by adjusting the significance threshold to $\propto =\frac{0.05}{12}$ (according to the total number of 12 mediator tests) ensuring that the findings reflect statistically significant effects not attributable to chance. The adjusted *α* is hence 0.0042. The presence of publication bias was assessed using funnel plots and further tested by Egger’s test. For the meta-analyses, further studies were excluded because they reported the prevalence relatively to comparison groups and did not provide prevalence numbers. We have set a limit of a minimal number of 10 available and qualitatively sufficient studies to conduct a meta-analysis. While we did not explicitly mention the minimum threshold of 10 studies in our preregistration, this criterion was implemented to ensure the reliability and validity of our results. This decision was guided by best practices in meta-analysis, which emphasize the importance of having enough data points to ensure that the results are statistically sound.

## 3 Results

### 3.1 Systematic review

This section provides a detailed overview of the prevalence, risk, and resilience factors for mental health conditions among female sex workers globally. The section is structured by conditions spanning from subclinical anxiety and anxiety disorders to depression, suicidality, PTSD, substance use, and dependence, as well as other conditions. Each sub-section reports the prevalence first (see Table 1) and the risk and resilience factors (see Table 2) second. Unless stated otherwise, the prevalences refer to current symptoms.

**Table 1:** Studies reporting the prevalence of mental conditions (anxiety, depression, PTSD, suicide and self-harm, substance use and dependence, obsessive-compulsive disorder, eating-disorder, somatization disorder, schizophrenia, other) meeting the inclusion criteria of the systematic review.

First author, year	Place	Sample size and population	Study design and recruitment strategy	Outcome variable and mental condition	Measurement	Main results
Abelson (2019) (25)	Cameroon	*N* = 2,165	Cross-sectional study (baseline data from intervention study), recruited through respondent driven sampling	Depression	The Patient Health Questionnaire (PHQ-9)	Depression was present among 49.8% (*n* = 1,067).
Alschech (2020) (89)	USA	*N* = 314; advertising their services online: 27.4%, selling sex in informal venues such as hrothels and clubs: 24.8%, selling sex on the street: 8%	Cross-sectional study, recruited online via E-Mail and from venues	PTSD	The PTSD Check List (PCL)	The study participants displayed a significant occurrence of symptoms indicative of post-traumatic stress, with an average PCL score of 45.82, which falls within the range suggesting a likely diagnosis of PTSD.
Beksinska (2021) (35)	Kenya	*N* = 1,003, 91.4% worked from a lodge/hotel/rented room	Baseline cross-sectional data from mixed method longitudinal study, recruited from seven specialized clinic services for sex workers	Anxiety	The Generalised Anxiety Disorder Tool (GAD-7)	38,4% of participants exhibited symptoms of anxiety (GAD-7 score ≥ 5), with 11% (95% CI: 9.3–13.1%) reporting moderate to severe anxiety
				Depression	The Patient Health Questionnaire (PHQ-9)	49.3% reported any symptom of depression (PHQ-9 score ≥ 5) with 3.2% (95% CI: 20.7–25.9%) reporting moderate/severe depression.
				PTSD	The Harvard Trauma Questionnaire (HTQ-17)	The prevalence of PTSD was 14.2% (95% CI: 12.2–16.5%)
				Substance use and dependence	The World Health Organization – Alcohol, Smoking and Substance Involvement Screening Test (WHO-ASSIST)	70.1% reported no or low risk alcohol use, while 29.9% reported moderate/high alcohol use. Regarding other substances (cannabis/amphetamine/cocaine/sedative/inhalants/hallucinogen), 69.3% reported no or low risk use, and 30.7%reported moderate/high risk substance use.
Beksinska (2022) (101)	Kenya	*N* = 1,003, 91,40% worked from a lodge/hotel/rented room	Baseline cross-sectional data from mixed method longitudinal study, recruited from seven specialized clinic services for sex workers	Substance use and dependence	The World Health Organization – Alcohol, Smoking and Substance Involvement Screening Test (WHO-ASSIST)	Harmful alcohol use was prevalent among 29.8 (95% CI: 27–32.6%). Harmful amphetamine (21.5%; 95% CI: 19.1–24.1%) and cannabis (16.8%; 95% CI: 14.7–19.2%) use were reported. 6.5% of women reported harmful use of both drugs as well as alcohol. Prevalence of other harmful drug use was low (sedatives 2.1%, cocaine 0.3%, opioids 0.5%, inhalants 0.1% and hallucinogens 0.1%). Only 0.5% of women reported injecting drug use in the last 3 months.
Bhardwaj (2023) (51)	eThekwiniSouth Africa	*N* = 1,384	Baseline data from adaptive intervention study, recruited by the peer case managers at known sex work venues	Depression	The Patient Health Questionnaire (PHQ-9)	33.2% were screened positive for depression.
				Substance use and dependence	The Alcohol Use Disorders Identification Test–Consumption (AUDIT-C)	40.1% were categorized as low risk. The moderate to high-risk category included 261 individuals (26.3%). The severe risk category consisted of 334 individuals (33.6%).
Carlson (2017) (61)	Ulaanbaatar, Mongolia	*N* = 222	Baseline data from intervention study for sex workers with alcohol use disorder, no recruitment strategy described	Depression	Depression symptoms were measured using the six-item depression subscale of the Brief Symptom Inventory (BSI)	60.4%, had a high risk for depression.
Chen (2017) (70)	Guangxi, Chongqing, Sichuan, and Xinjiang, China	*N* = 457	Cross-sectional study, recruited from different venues	Depression	Center for Epidemiological Studies Depression Scale (CES-D)	The prevalence of depression among female sex workers was 41.3%.
Chudakov (2002) (88)	Be′er Scheva, Israel	*N* = 55	Cross-sectional part of a mixed methods study, recruited via newspaper ads, escort agencies and in brothels	Depression	The Center for Epidemiologic Studies Depression (CES-D) Scale	33% had an average depression score of over 2.5 (indicating presence of depressive symptoms), 19% had averages over 3 (likely to be clinically depressed).
				PTSD	PTSD symptoms using the PTSD checklist (PCL)	17% of the women showed symptoms of PTSD.
Cigrang (2020) (84)	Dayton, Ohio, USA	*N* = 91 sex workers who were arrested for at least one drug offense	Data from Intervention Study, recruited from jail	Depression	The Patient Health Questionnaire (PHQ-9)	54% of participants exhibited symptoms of moderate to severe major depression.
				PTSD	The Primary Care–Posttraumatic Stress Disorder Screen (PC-PTSD)	66% of participants screened positive for PTSD according to the PC-PTSD. Those who reported engaging in sex trading over the past 12 months had significantly higher PHQ-9 scores, averaging 14.7, compared to 11.4 for those who did not report such history.
				Substance use and dependence	The Alcohol Use Disorders Identification Test–Consumption (AUDIT-C), The Drug Abuse Screening Test–10 (DAST-10)	35% met the cut-off score for hazardous drinking. 73% displayed severe drug problems.
Coetzee (2018) (67)	Soweto, South Africa	*N* = 508	Cross sectional study, recruited through respondent driven sampling	Depression	The Center for Epidemiologic Studies Depression Scale (CES-D)	The median score for depressive symptoms was 25 (IQR: 19 ± 31), with 68.7% having high depression symptoms.
				Suicide and self-harm	The Center for Epidemiologic Studies Depression Scale (CES-D)	The majority of female sex workers (85.6%) reported no suicidal ideation or attempts in the past 12 months. Suicidal ideation (but no attempts) were reported by 3.6%, while 9.8% reported ideation and attempts (1% reported attempting suicide but without ideation).
				PTSD	The Posttraumatic Stress Disorder - 8 items (PTSD-8)	The median PTSD symptom score was 18 (IQR: 10 ± 22). PTSD diagnostic criteria were met in 39.6% of participants.
				Substance use and dependence	Alcohol Use Disorders Identification Test Consumption (AUDIT-C)	54% of those affected by depression engaged in severe binge drinking. Severe binge drinking was found amongst 57.6% of those suffering from PTSD.
Couture (2016) (93)	Preah Sihanouk province, Cambodia	*N* = 100 female sex workers, *N* = 100 male clients	Cross-sectional, recruited at entertainment/drinking establishments and other sex work venues	Substance Use and Dependence	The Alcohol Use Disorders Identification Test Consumption (AUDIT-C) and PEth (biomarker for alcohol abuse) using the Whatman no. 903; Whatman International, Maidstone, UK test kit	A significant majority (85%) of female sex workers reported unhealthy alcohol use based on AUDIT-C results. Furthermore, 60% had PEth biomarker levels indicating alcohol consumption (≥50 ng/mL). Combining both AUDIT-C and PEth markers, 88% of the women either had a PEth level of at least 50 ng/mL or tested positive on the AUDIT-C. While only 30% reported drinking four or more times a week in the past 3 months, 76% consumed five or more drinks on a typical drinking day. Additionally, 81% had been intoxicated, and 83% engaged in recent heavy episodic drinking.
Decker (2022) (40)	Mae Sot, Thailand	*N* = 128, migrant sex workers	Mixed methods study including a cross-sectional survey, recruited by respondent driven sampling	Anxiety	The Hopkins Symptoms Checklist-25 (HSCL-25)	Anxiety affected 75.8%.
				Depression	The Hopkins Symptoms Checklist-25 (HSCL-25)	87.5% reported depression.
Edwards (2006) (26)	Chapel Hill, North Carolina, USA	*N* = 669 African American Crack Abusers, of whom *N* = 295 (44.10%) reported trading sex in the past 30 days	Intervention study (baseline data)	Anxiety	Drug Abuse Treatment AIDS Risk (DATAR) Anxiety Scales	For individuals who engaged in trading sex, the mean anxiety score was 13.4, with a standard deviation of 6.1. In contrast, those who did not engage in trade sex had a lower mean anxiety score of 10.8, with the same standard deviation of 6.1. A statistically significant difference was observed between the two groups.
				Depression	Drug Abuse Treatment AIDS Risk (DATAR) Depression Scales	For those who have traded sex, the mean depression score was 14.2 (SD = 4.8). For those who have not traded sex, the mean depression score was lower, at 11.8 (SD = 4.8), (*p* < 0.05).
				PTSD	Items from the Global Appraisal of Individual Needs (GAIN)	Women who trade sex had a mean PTSD score of 21.1 (SD = 10.1). In contrast, women who do not trade sex had a lower mean PTSD score of 16.2 (SD = 10). This difference was statistically significant (*p* < 0.05).
				Substance Use and Dependence	The Revised Risk Behavioral Assessment (RRBA), Crack use was coded as heavy use (>15 days) or less heavy use (≤15 days) based on the number of days of crack use in the past 30 days	A higher percentage of women engaged in sex work used crack cocaine heavily in the last 30 days compared to women not involved in sex work (66.8 vs. 34.5%, *p* < 0.05).
Edwards (2016) (117)	Midwest, USA	*N* = 171 female offenders, *N* = 319 drug users, of whom *N* = 135 were female sex workers	Cross-Sectional Study, recruited from jail, community centers and through advertisements and flyers	Substance Use and Dependence	SCID-I for DSM-IV-TR	Drug dependence (OR = 1.26, 95% CI [0.91, 1.74], *p* = 0.17) was not significantly related to direct sex work.
				Other	Psychopathy Checklist: Screening Version (PCL:SV)	PCL: SV score (OR = 1.13, 95% CI [0.98, 1.30], *p* = 0.10) was not significantly related to sex work.
Fang (2007) (75)	Guangxi, China	*N* = 454	Cross sectional study, recruited from restaurants, barbershops, and hair-washing rooms from three geographic locations in the County	Suicide and Self-Harm	Suicide ideation and attempt according to ICD-10 criteria	Approximately 14% of the female participants considered suicide in the past 6 months. 8% had made suicide attempts within the same period.
Farley (2016) (86)	Minneapolis, Duluth, and Bemidji, Minnesota, USA	*N* = 105 American Indian/Alaska Native (AI/AN) women in sex work from street prostitution (85%); private residences (83%); private parties, hotels, or nightclubs (69%); and bars (68%)	Cross-sectional study, recruited through advertisements and via snowball or chain referral sampling	PTSD	The Dissociation subscale of Briere’s Trauma Symptom Checklist (TSC-40), the Post-traumatic Stress Disorder Checklist (PCL)	70% of the women met criteria for flashbacks, 61% met criteria for avoidance and numbing, and 74% met criteria for autonomic nervous system hyperarousal. 52% percent met all criteria for a diagnosis of PTSD; their mean PTSD severity score was 51 (SD = 19).
Ghafoori (2023) (43)	California, USA	*N* = 1,264, *N* = 103 woman who experienced sex trafficking (ST), *N* = 606 who experienced domestic violence (DV), and *N* = 555 who experienced sexual assault (SA)	Secondary data analysis of data gathered from a community mental health treatment clinic for individuals who experienced violence	Anxiety	The Brief Symptom Inventory-18 (BSI-18)	Although many ST survivors reported screen positive for anxiety (59.6%), the proportion of participants reporting these conditions were significantly lower compared with DV (70%), and SA survivors (79.3%)
				Depression	The Brief Symptom Inventory-18 (BSI-18)	Although 60.6% of ST survivors reported screen-positive depression, this proportion was significantly lower compared to DV survivors, who had a 73.6% depression rate, and SA survivors, who had a 74.6% depression rate.
				PTSD	The Life Events Checklist forDSM-5 (LEC-5), The PTSD Checklist for DSM-5 (PCL-5)	Although many ST survivors reported screen positive PTSD (61.2%), the proportion of participants reporting the condition were significantly lower compared with DV (70.5% PTSD) and SA survivors (83.6% PTSD).
Gilchrist (2005) (42)	Glasgow, Scotland	*N* = 176 Drug-Using Sex Workers, *N* = 89 Drug-Users (no sex workers)	Cross-sectional study with control group, recruited via convenience sampling from a drop-in center, a crisis center and a methadone medical specialist service	Anxiety	The Revised Clinical Interview Schedule (CIS-R)	60% of sex workers reported anxiety compared to 53% of non-sex-workers. 24% of female sex workers experienced panic disorder compared to 29% of non-sex-workers. 26% of female sex workers reported phobias compared to 30% of non-sex-workers. However, group differences were not significant.
				Depression	The Revised Clinical Interview Schedule (CIS-R)	For depression, 70% of the sex workers (123 out of 176) exhibited symptoms, compared to 61% of the non-sex workers (54 out of 89). However, the difference was not statistically significant.
				Suicide and Self-Harm	The Revised Clinical Interview Schedule (CIS-R)	39% of sex workers (69 out of 176) engaged in deliberate self-harm, compared to 30% of non-sex workers (27 out of 89). The difference between both groups was not significant. 53% of sex workers (93 out of 176) had attempted suicide, compared to 39% of non-sex workers (35 out of 89).
				Substance Use and Dependence	The Diagnostic Interview Schedule (DIS) assessed 12-month drug use	79% (139/176) of sex workers and 84% (75/89) of non-sex workers reported poly drug use in the last 30 days, with an OR (95% CI) of 0.70 (0.36–1.38). 18% (31/176) of sex workers and 7% (6/89) of non-sex workers reported cocaine use in the last 30 days, with an OR (95% CI) of 2.96 (1.18–7.38). 72% (127/176) of sex workers and 51% (45/89) of non-sex workers reported injecting drugs in the last 30 days, with an OR (95% CI) of 2.53 (1.49–4.31). Accidental Drug Overdose: 55% (97/176) of sex workers and 27% (24/89) of non-sex workers experienced accidental drug overdose, with an OR (95% CI) of 3.32 (1.91–5.79). 12-month heroin dependence was reported by 96% (169/176) of sex workers and 97% (86/89) of non sex workers. 12-month illicit tranquillizer dependence was reported by 49% (87/176) sex workers and 49% (44/89) of non sex workers.
				Obsessive-compulsive disorder	The Revised Clinical Interview Schedule (CIS-R)	37% of female sex workers (66 out of 176) reported experiencing compulsions, versus 34% of non-sex workers (30 out of 89). No significant group difference could be observed. 53% of female sex workers (94 out of 176) reported having obsessions, compared to 47% of non-sex workers (42 out of 89). The group difference was not statistically significant.
				Eating-disorder	The Revised Clinical Interview Schedule (CIS-R)	Among female sex workers, 37% (64 out of 175) reported having experienced an eating disorder at some point in their lives, compared to 27% of non-sex workers (24 out of 89). There was no significant difference between both groups.
Griffith (2013) (102)	Los Angeles, California, USA	*N* = 176 Performers in the adult entertainment industry, 58 performers (32.90%) identified as heterosexual and 118 (6710%) identified as bisexual	Cross-sectional, convenience sampling conducted at the Adult Industry Medical Healthcare Foundation (AIM)	Substance Use and Dependence	The Short Michigan Alcohol Screening Test (SMAST), The Texas Christian University (TCU) Drug Screen	The SMAST scores did not show a significant difference between bisexual porn actresses (M = 1.44, SD = 2.15) and heterosexual porn actresses (M = 1.39, SD = 1.55), with both groups having mean scores <2. A score of 3 or higher on the SMAST indicates an alcohol problem. The findings in this study suggest that levels of problem drinking among porn actresses are similar to those in the general population. Marijuana: 83.9% of bisexual actresses vs. 69% of heterosexual actresses Hallucinogens: 41.4% of bisexual actresses vs. 24.6% of heterosexual actresses Heterosexual porn actresses used cocaine more frequently (M = 1.0, SD = 0.93) than bisexual actresses (M = 0.53, SD = 0.74)
Grudzen (2011) (53)	California, USA	*N* = 134 Adult Film Performer *N* = 1773 Women of similar age	Cross-sectional structured online survey, recruited via Email	Depression	The Patient Health Questionnaire–8 (PHQ-8)	33% of performers met the criteria for current depression based on PHQ-8 scores, compared to 13% of similar-aged California women (*p* < 0.01).
Hendrickson (2023) (71)	Baltimore, Maryland, USA	*N* = 370 Street-Based Sex Workers	Baseline, 6-month, and 12-month follow-up data from interventional study, recruited using time–location sampling	Depression	The Patient Health Questionnaire (PHQ-9)	Depression was found in 46% of the total sample. A larger percentage of women with high residential mobility had more severe depressive symptoms than did their less-mobile counterparts (51 vs. 40%, respectively; *p* = 0.037).
				PTSD	The PTSD Checklist for DSM-5 (PCL-5)	53% had high PTSD symptoms based on the PCL-5.
Hong (2013) (54)	Guangxi, China	*N* = 1,022	Cross-sectional survey, recruited from 60 establishments that represented all known commercial sex venues	Depression	The Center for Epidemiologic Studies Depression scale (CES-D)	The mean score for depression, as measured by the CES-D, was 17.12 with a standard deviation of 9.65. In total 49% suffered from depression.
Iaisuklang (2017) (41)	Shillong, Meghalaya, India	*N* = 100	Cross-sectional study, a simple random sampling procedure was followed	Anxiety	The Mini International Neuropsychiatric Interview (M.I.N.I.)	8% of participants have been diagnosed with General Anxiety Disorder.
				Depression	The Mini International Neuropsychiatric Interview (M.I.N.I.)	Nine participants (9.0%) were experiencing a current major depressive episode. In the past, 25 participants (25%) have experienced a major depressive episode. Additionally, 3 participants (3%) were currently experiencing a major depressive episode with melancholic features.
				PTSD	The Mini International Neuropsychiatric Interview (M.I.N.I.)	21% have been diagnosed with PTSD
				Substance Use and Dependence	The Mini International Neuropsychiatric Interview (M.I.N.I.)	8% have been diagnosed with alcohol dependence. 85% reported current tobacco use. 25% did not use tobacco. 12% reported using cannabis. 82% did not use cannabis. 3% have been diagnosed with a non-alcohol psychoactive substance use disorder.
				Other	The Mini International Neuropsychiatric Interview (M.I.N.I.)	9% have been diagnosed with antisocial personality disorder.
Jiwatram-Negron (2015) (123)	New York City, USA	*N* = 346 Women, *N* = 97 Sex Workers	Baseline data from RCT, recruited via street outreach, homeless shelters, soup kitchens, word of mouth, and syringe exchange programs. Most couples entered the study through street outreach	Other	Multi-dimensional Scale of Perceived Social Support (MSPSS), Brief Symptom Inventory (BSI), the Global Severity Index (GSI)	Overall, 30% of participants reported being hospitalized for mental health issues in their lifetime, with significantly more sex traders reporting prior hospitalization (46%) compared to non-trading women (24%; *p* < 0.01). Using the Global Severity Index (GSI) of the Brief Symptom Inventory (BSI), similar patterns emerged: although the average GSI score was 0.7, women engaged in sex trading reported greater psychological distress than nontrading women (0.95 and 0.6, respectively; *p* < 0.01)
Jung (2008) (87)	Korea	*N* = 113 Former Sex Workers *N* = 81 Activists, *N* = 111 Control Group	Cross-sectional study, recruited from 18 shelters	Depression	The Stress Response Inventory (SRI)	Activists showed greater scores in depression than the other 2 groups. Compared to the control group, the activists group also had a higher mean total score of SRI and, particularly in the scales of depression and tension. There was no significant difference between activists and former sex workers.
				PTSD	The Davidson Trauma Scale (DTS), The modified Impact of Event Scale-Revised (IES-R)	Former sex workers exhibited a higher prevalence of PTSD symptoms compared to activists, who in turn reported higher scores than the control group (*p* < 0.001).
Kelton (2023) (44)	35 U.S. States and Puerto Rico	*N* = 102 Stripper	Cross-sectional study, recruited via snowball sampling	Anxiety	the Generalized Anxiety Disorder Scale-7 (GAD-7)	Participants reported an average score of 6.75 (SD = 5.05) on the GAD-7 for current anxiety symptoms, indicating clinically significant distress within the mild severity range.
				Depression	The Patient Health Questionnaire-8 (PHQ-8)	Regarding current depressive symptoms, participants reported an average score of 12.86 (SD = 6.64) on the PHQ-8. This score is above the clinical significance threshold and is consistent with symptoms of major depressive disorder.
Kurtz (2005) (104)	Miami, USA	*N* = 586	Mixed-methods with a cross-sectional study design as quantitative part, recruited via snowball sampling	Substance Use and Dependence	The Revised Risk Behavioral Assessment (RRBA)	76.1% reported Alcohol Use, 73.4% reported Crack cocaine use, 59.9% reported Marijuana use, 42.8% reported Other cocaine use, 19.5% reported Heroin use and 13.5% reported injecting any drug.
Kim (2020) (58)	Chicago, Illinos, USA	*N* = 400 (*N* = 181 sex workers, Care/Service work only *N* = 92, Engaged in both *N* = 68)	Cross-sectional study, recruited from jail	Depression	The Center for Epidemiologic Studies Depression Scale (CES-D)	Women engaged in care/service work only were less likely to have clinical depression (64.8%) compared with women in sex work only (81%) and women working in both job types (82.1%). Women engaged exclusively in care/service work were less likely to have clinical depression (*p* < 0.05).
Lichtwarck (2023) (100)	Dar es Salaam, Tanzania	*N* = 470	Baseline data from a quasi-experimental intervention study, recruited participants using respondent driven sampling (RDS)	Substance Use and Dependence	The Alcohol Use Disorders Identification Test (AUDIT)	Out of 405 participants (86.2%) who reported using alcohol in the year preceding the survey: 360 (69.6%) had an AUDIT score of 8 or above, indicating “hazardous alcohol use.” 230 (37.3%) had an AUDIT score of 16 or more, suggesting “harmful alcohol use” or “alcohol dependency.” Additionally, more than half of the entire sample (296 participants; 53.6%) had been drinking alcohol the last time they had sex with a client.
Ling (2007) (74)	Hong Kong, China	*N* = 89 Street-Based Sex Workers	Cross-sectional study recruited subjects through outreach visits	Suicide and Self-Harm	Suicide ideation and attempts according to ICD-10 criteria	In this study, 25.8% of participants reported having either attempted or contemplated suicide. Specifically, 6.7% admitted to attempting suicide, with 2.3% trying at least twice.
MacLean (2018) (65)	Lilongwe, Malawi	*N* = 200	Cross-sectional study, venue-based sampling at 23 establishments through outreach team	Depression	The Patient Health Questionnaire (PHQ-9)	The average score on the PHQ-9 was 4.7, with a standard deviation of 3.4. The median score was 5, with an interquartile range of 2–7. The rate of likely depression (PHQ-9 score of 10 or higher) was 8%, with 7% showing moderate depressive symptoms and 1% displaying moderate-to-severe symptoms. Additionally, 43% of female sex workers had mild depression (PHQ-9 scores of 5–9), which was not significant enough to indicate a probable clinical diagnosis.
				PTSD	The PTSD Check List – Civilian Version (PCL-C)	The PTSD prevalence rates were identified as 16% (score over 38) and 10% (score over 44), in total 8% met the criteria for probable PTBS.
Maclin (2023) (23)	Santo Domingo, DominicanRepublic	*N* = 211 cisgender female, *N* = 101 transgender female sex workers living with HIV	cross-sectional surveys, baseline data from intervention study, recruited via non-random sampling led by peer navigators	Anxiety	The anxiety-specific module from the Hospital Anxiety and Depression Scale (HADS-A)	In the cisgender group 85 individuals (40.3%) scored as Borderline Abnormal or Abnormal on the HADS-A, while 126 individuals (59.7%) scored normal. In the transgender group (*N* = 100), 34 individuals (34%) scored as Borderline Abnormal or Abnormal on the HADS-A, and 66 individuals (66%) scored as Normal. There were no significant differences between the two groups.
				Depression	The Patient Health Questionnaire-9 (PHQ-9)	25.5% (54 individuals) experienced moderate to severe depression, as indicated by scores of 10 or higher. In contrast, 74.4% (157 individuals) exhibited minimal to mild depression, with scores of 9 or lower. There was no significant difference between both groups.
Markova (2019) (78)	Ukraine	*N* = 300	Cross-sectional study, recruited through Telephone from the database of The AIDS Support Organization (TASO)	Suicide and Self-Harm	The Colombian scale for assessing the severity of suicide (C-SSRS)	Feeling suicidal without concrete plans was reported by 34.1%. 21.5% had concrete suicide plans. 41.5% tried to commit suicide by making a real attempt.
Murnan (2018) (68)	Columbus, Ohio, USA	*N* = 183 Sex Workers who were Mothers with Substance Use Disorder	Randomized Control Trial, recruited from a large substance use treatment facility	Depression	The Beck Depression Inventory II (BDI-II)	Two programs were tested against each other in regard to decreasing depression rates. EBFT: Ecologically-Based Family Therapy and WHE: Women’s Health Education, which is a 12-session psycho-educational intervention that focuses on topics such as female anatomy, human sexual behavior, pregnancy and childbirth, and sexually transmitted infections. EBFT showed to be effective to reduce depression rates.
				Substance Use and Dependence	The Form-90	The EBFT home group showed significantly less moderate and severe substance use compared to the WHE group. EBFT (Ecologically-Based Family Therapy) and WHE (Women’s Health Education) are interventions, with WHE focusing on topics such as female anatomy, human sexual behavior, pregnancy and childbirth, and sexually transmitted infections.
Nabayinda (2023) (29)	Southern Uganda	*N* = 542	Baseline data from interventional study, recruited from 19 HIV hot spots across four districts in Southern Uganda	Depression	The six-item scale from the Brief Symptoms Inventory Survey (BSI)	The mean depression score was 10.91 with a standard deviation of 4.98 (on a scale from 7 to 35).
Nuttbrock (2004) (103)	New York City, USA	*N* = 179 Street-Based Sex Workers	Intervention Study (baseline data), recruited via outreach program	Substance Use and Dependence	The recent use of cocaine and opiates was also assessed biologically using radioimmunoassays of hair.	92.7% of the women were found to have used cocaine, and 44.4% had used opiates in the month preceding the test. The mean severity of dependence during the prior month was 11.32 for heroin, 7.3 for crack cocaine, 6.24 for alcohol, 3.84 for powder cocaine, 3.36 for benzodiazepines (including barbiturates and other depressants), and 2.63 for marijuana/hashish.
Ostrovschi (2011) (49)	Moldova	*N* = 178, *N* = 120 at follow-up, Survivors of Human Trafficking	Longitudinal study, recruited either face to face or by phone, during their monthly follow-up appointment in the rehabilitation programme	Anxiety	clinical interview following ICD-10 criteria, at follow up: Structured Clinical Interview for DSM Disorders (SCID), according to DSM-V criteria	Initially, in the crisis intervention phase (1–5 days after return), 4 individuals (3.3%) were identified with panic disorder. This number slightly increased to 5 individuals (4.1%) in the re-integration phase (2–12 months after return). During the crisis intervention phase, 4 individuals (3.3%) were diagnosed with generalized anxiety disorder. This number doubled to 8 individuals (6.6%) during the re-integration phase.
				Depression	clinical interview following ICD-10 criteria, at follow up: Structured Clinical Interview for DSM Disorders (SCID), according to DSM-V criteria	During the crisis intervention phase, 4 individuals (3.3%) were diagnosed with major depression, which significantly increased to 24 individuals (16.7%) during the re-integration phase.
				PTSD	Clinical interview following ICD-10 criteria, at follow up: Structured Clinical Interview for DSM Disorders (SCID), according to DSM-V criteria	During the crisis intervention phase, 58 individuals (48.3%) were diagnosed with PTSD. In the re-integration phase, the number decreased to 43 individuals (35.8%).
				Substance use and dependence	Alcohol Use Disorders Identification Test (AUDIT) scale	Among women with psychiatric illness during the crisis intervention phase, 5 (4.2%) exhibited harmful alcohol use (according to ICD-10). Among women with psychiatric illness during the re-integration phase, 10 (8.3%) exhibited alcohol abuse (according to DSM-5). In the first phase, 8 women (7%) showed alcohol dependence, while in the second phase, 5 women (4.2%) showed signs of dependence. During the crisis intervention phase, 4 individuals (3.3%) were identified with Substance Harmful Use/Abuse. In the re-integration phase, the number slightly increased to 5 individuals (4.2%).
				Somatization disorder	Clinical interview following ICD-10 criteria, at follow up: Structured Clinical Interview for DSM Disorders (SCID), according to DSM-V criteria	In the crisis intervention phase, 3 individuals (2.5%) were diagnosed with Somatization Disorder. In the re-integration phase, there were no individuals (0%) diagnosed with Somatization Disorder.
				Schizophrenia	Clinical interview following ICD-10 criteria, at follow up: Structured Clinical Interview for DSM Disorders (SCID), according to DSM-V criteria	During the crisis intervention phase, 2 individuals (1.6%) were diagnosed with Paranoid Schizophrenia. In the re-integration phase, there were no individuals (0%) diagnosed with Paranoid Schizophrenia.
				Other	Clinical interview following ICD-10 criteria, at follow up: Structured Clinical Interview for DSM Disorders (SCID), according to DSM-V criteria	During the crisis intervention phase, 16 individuals (13.3%) were identified with Adjustment Disorder. By the re-integration phase, there were no individuals (0%) reported to have Adjustment Disorder. Initially, in the crisis intervention phase, 12 individuals (10%) were diagnosed with Acute Stress Reaction. This number also reduced to zero (0%) in the re-integration phase.
Ouma (2021) (69)	Northern Uganda	*N* = 300	Cross-sectional study, recruited through Telephone from the database of The AIDS Support Organization (TASO)	Depression	The Mini International Neuropsychiatric Interview (M.I.N.I.)	The prevalence of major depression among female sex workers was 47.7%. Among these, 91% had either severe (50.4%) or moderate (40.6%) depressive symptoms, with only 9% experiencing mild symptoms.
Pandiyan (2012) (126)	Bangalore, India	*N* = 100	Cross-sectional study, recruited at the psychiatry outpatient department of the Victoria hospital, Bangalore	Depression	Clinical interview following ICD-10 criteria	71% were suffering from depression.
				Substance use and dependence	The alcohol use disorder identification test (AUDIT)	In the study population all were using alcohol. 74% were using tobacco, 6% cannabis, 14% opioids, and 6% cocaine.
Patel (2015) (59)	Andhra Pradesh, Southern India	*N* = 1986	Cross-sectional survey, probability sampling (for public venues) and conventional cluster sampling (for non-public venues) methods were used (sex workers were then recruited from each hot spot)	Depression	The Patient Health Questionnaire-2 (PHQ-2) depression scale	60.8% of respondents (1,208 individuals) showed no or low signs of depression. 39.2% (778 individuals) were categorized as having major depression.
Rash (2016) (95)	Farmington, New Mexico, USA	*N* = 493 Women with Cocaine Use Disorder, of whom *N* = 215, 43.60% were Sex Workers	Baseline Data from Intervention Study, recruited from outpatient psychosocial treatment clinics	Substance Use and Dependence	The Structured Clinical Interview for the DSM-IV (SCID), Urine samples for opioids and cocaine using Ontrak TesTstiks (Roche, Somersville, NJ, USA), the Addiction Severity Index (ASI)	56.1% of women without a history of sex trade and 60.9% of those with a history of sex work were found to have Alcohol Use Disorder. The statistical analysis indicated no significant difference in prevalence between the two groups. Among Non-Sex Workers 79 individuals (28.4%) had positive urine samples while among Sex Workers 52 individuals (24.3%) had positive urine samples. The Chi-square test result indicated no significant difference in the rate of drug-positive samples between non-sex workers and sex workers. Among non-sex workers, 80 individuals (28.9%) had opioid use disorder, while among sex workers, 61 individuals (28.5%) had opioid use disorder. The p-value (*p* = 0.93) indicates no significant difference between the two groups.
Risser (2006) (33)	Houston, Texas, USA	*N* = 193 African American Crack Cocaine Users who currently (*N* = 128), previously (*N* = 26), or never (*N* = 39) traded sex for money	Baseline data from intervention study, recruited using targeted sampling and participant referral in neighborhoods with high rates of drug use	Anxiety	TCU Psychosocial Functioning and Motivation Scales	Current traders had a mean anxiety score of 3.4 with an SD of 0.78. Previous traders had a mean score of 3.2 with an SD of 0.85, while never traders had the lowest mean score of 2.7 with an SD of 0.71. The *P*-value for the trend across these groups was <0.001.
				Depression	TCU Psychosocial Functioning and Motivation Scales	Current sex workers were more likely to report more depression (Mean: 3.3 SD: 0.76) compared to never sex traders (Mean 2.6 SD: 0.67 *p* ≤ 0.001). However, there were no significant differences between current and previous sex workers.
				Substance Use and Dependence	Peer Outreach Questionnaire (PRQ), urine sample tested for cocaine metabolites using ONTRACK test kits (Varian, Inc.)	Current traders had been using crack cocaine for a longer duration, with the longest durations reported by current and former traders (10.1 and 10.2 years, respectively), compared to 6.5 years for those who never traded (*p* < 0.005). Current traders reported a significantly higher frequency of crack use in the past 30 days, with usage frequencies of 93 times for current traders, 60 times for former traders, and 51 times for those who never traded (*p* = 0.02).
Rössler (2010) (16)	Zurich, Switzerland	*N* = 193	Cross-sectional study, utilized quota sampling	Anxiety	The Composite International Diagnostic Interview (M-CIDI 2.1)	Generalized Anxiety Disorder had a one-year prevalence of 5.2% (± 3.3) and a lifetime prevalence of 7.3% (± 3.8). Panic Disorder had a one-year prevalence of 8.8% (± 4.1), and a lifetime prevalence of 11.4% (± 4.6). Simple Phobia showed a one-year prevalence of 17.6% (± 5.4), and a lifetime prevalence of 18.7% (± 5.5). Social Phobia had both a one-year and lifetime prevalence of 7.3% (± 3.8). Agoraphobia without panic had a one-year prevalence of 2.1% (± 2) and a lifetime prevalence of 3.1% (± 2.8). Anxiety Disorder had a one-year prevalence of 1% (± 2) and a lifetime prevalence of 2.1% (± 2.4).
				Depression	The Composite International Diagnostic Interview (M-CIDI 2.1)	For major depression, the one-year prevalence rate was 24.4% (95% CI: +/− 6) and the lifetime prevalence rate was higher at 36.3% (95% CI: +/− 6.7). Dysthymia showed a one-year prevalence rate of 11.9% (95% CI: +/− 4.7), the lifetime prevalence was slightly higher at 12.4% (95% CI: +/− 4.70). For bipolar disorders, both the one-year and lifetime prevalence rates were 0.5%, with a 95% confidence interval (CI) standard deviation (±) of 1.
				PTSD	The Composite International Diagnostic Interview (M-CIDI 2.1)	For PTSD, the one-year prevalence was 13%, with a 95% confidence interval (CI) standard deviation (±) of 4.8. The lifetime prevalence was higher at 21.2%, with a standard deviation (±) of 5.8.
				Substance Use and Dependence	The Composite International Diagnostic Interview (M-CIDI 2.1)	For alcohol dependency, the one-year prevalence was reported as 0%. For the lifetime prevalence, it was 0.5%, with a 95% confidence interval (CI) standard deviation (±) of 1.
				Schizophrenia	The Composite International Diagnostic Interview (M-CIDI 2.1)	Both the one-year and lifetime prevalence rates are reported as 0%.
				Somatoform disorder	The Composite International Diagnostic Interview (M-CIDI 2.1)	For somatoform disorders, the one-year prevalence was 10.4%, with a 95% confidence interval (CI) standard deviation (±) of 4.4. The lifetime prevalence slightly increased to 11.4%, with a standard deviation (±) of 4.6.
				Obsessive-compulsive disorder	The Composite International Diagnostic Interview (M-CIDI 2.1)	For obsessive-compulsive disorder, both the one-year and lifetime prevalence rates were reported at 2.1%, with a 95% confidence interval (CI) standard deviation (±) of 2.4
				Eating-disorder	The Composite International Diagnostic Interview (M-CIDI 2.1)	The one-year prevalence is 5.2%, with a standard deviation (±) of 3.3.The lifetime prevalence increased to 8.8%, with a standard deviation (±) of 4.1.
Rossouw (2021) (52)	Nelson Mandela Bay Municipality, South Africa	*N* = 410	Cross-sectional recruited via respondent driven sampling	Depression	The Patient Health Questionnaire-9 (PHQ-9)	More than half of the participants were not depressed, as indicated by a PHQ-9 score of <5 [55.9, 95% Bayesian Confidence Interval (BCI) 49–62.8]. Approximately 30% of female sex workers in the sample, and 28.8% (95% BCI 22.4–35.2) of female sex workers in the Nelson Mandela Bay area, had clinically significant depression, according to a PHQ-9 cutoff score of more than 9.
Roxburgh (2006) (9)	Sydney, Australia	*N* = 72 Street-Based Sex Workers	Cross sectional study, recruited through agencies	Depression	The Beck Depression Inventory-II (BDI-II)	Most participants in the study (87%) reported experiencing depressive symptoms ranging from mild to severe. Additionally, over half of the participants (54%) indicated experiencing severe current depressive symptoms.
				Suicide and Self-Harm	The National Mental Health and Wellbeing (NHMWB) version of the Composite International Diagnostic Interview (CIDI)	74% of the sample reported having ever thought about suicide, and just under half (42%) reported having made a suicide attempt.
				PTSD	The National Mental Health and Wellbeing (NHMWB) version of the Composite International Diagnostic Interview (CIDI)	47% of the sample met DSM-IV criteria for a lifetime diagnosis of PTSD. Among those with PTSD, 91% had chronic symptoms lasting 3 months or longer, and 82% reported that their symptoms lasted for 1 year or more. The median time since the most stressful traumatic event for those with PTSD was 17 years (range 1–52 years). Despite this, 62% of those with a lifetime diagnosis of PTSD (31% of the sample) also met DSM-IV criteria for current PTSD (within the preceding 12 months).
				Substance Use and Dependence	Severity of Dependence Scale (SDS)	94% reported ever injecting any drug, 23% reported injecting before age 16.82% reported Heroin dependency, 36% reported Cocaine dependency, 32% were Cannabis dependent.
Shahmanesh (2009) (79)	Goa, India	*N* = 326	Cross-sectional study, recruited in cooperation with the largest HIV non-governmental organization in Goa via respondent driven sampling	Suicide and Self-Harm	the Kessler 10 (K10)	During the past 3 months, the prevalence of suicidal ideation was 34.9% (95% CI: 29.8%-40.3%; *n* = 126), the prevalence of suicide planning was 25.6% (95% CI: 21.1%-30.6%; *n* = 95), and the prevalence of suicide attempts was 18.70% (95% CI: 14.9%-23.3%; *n* = 73). Among women younger than 20 years, the prevalence of suicide attempts in the past 3 months was 41.5% (*n* = 17).
Sherwood (2024) (50)	Pattaya, Thailand	*N* = 232 Venue-Based Sex Workers	Secondary data analysis from a quasi-experimental study, recruited via venue-based sampling from two sex work zones/hotspots	Depression	The two-question Patient Health Questionnaire (PHQ-2)	10.3% of the sample displayed depression at baseline.
Slim (2020) (34)	Lebanon	*N* = 60 Sex Workers, *N* = 60 Non Sex Workers	Case–control study, recruited from prison	Anxiety	Hamilton Anxiety Rating Scale (HAM-A)	Anxiety scores were significantly higher in sex workers (12, 23) compared to non-sex workers (21, 24). *p* < 0.05
				Depression	The validated Arabic version of the Hamilton depression rating scale (HDRS) was used in this study	Sex workers scored higher on the depression scale (24.05 vs. 9.75) than non sex workers. *p* < 0.05
Stockton (2020) (63)	Nairobi, Kitui, Busia, and Homa Bay, Kenya	*N* = 497 female sex workers, *N* = 232 male sex workers	Cross-sectional survey, recruited via snowball sampling	Depression	The Patient Health Questionnaire (PHQ-9)	The prevalence of depression was 39.4% among female sex workers and 22% among male sex workers.
Stoebenau (2023) (91)	eThekwini, South Africa	*N* = 644, 38.50% reported no exchange sex in the past year, 17.60% only transactional sex with a main partner (but no other form of exchange sex), 34.50% transactional sex with a casual partner (but not sex work), and 9.50%, 61 participants were engaged in sex work	Baseline data from intervention study (RCT), recruited through convenience sampling from 34 clusters	Depression	The Centre for Epidemiologic Studies Depression Scale (CES-D)	62.3% of sex workers experienced depression (CI: 49.5–73.6%), with an aOR of 2.09 (CI: 1.14–3.86, *p*-value: 0.018), indicating a statistically significant increase in the odds of depression compared to the reference group.
				Substance Use and Dependence	Alcohol Use Disorders Identification Test (AUDIT) scale	28 individuals, 45.9% (95% CI: 33.9–58.4) showed problematic alcohol use.
Suresh (2009) (85)	Chennai, India	*N* = 57 Street-Based Sex Workers	Cross-sectional study, recruited randomly through an agency	Depression	The Center for Epidemiologic Studies Depression (CES-D) Scale	No participants reported experiencing depressive symptoms for less than a day per week. 14% experienced depressive symptoms 1 to 2 days per week, 60% experienced symptoms 3 to 4 days per week, and 26% experienced symptoms 5–7 days per week. On average, participants reported feeling depressed 3.10 days per week, with a standard deviation of 0.63 days.
				PTSD	The PTSD Check List – Civilian Version (PCL-C)	None of the participants (0%) reported experiencing PTSD “not at all.” 42% reported experiencing PTSD to a “moderate” extent. 35% indicated experiencing PTSD “quite a bit,” and 12% reported experiencing PTSD to an “extreme” extent. Overall, 89% of participants reported experiencing moderate to extreme PTSD.
Tchankoni (2020) (94)	Togo, Africa	*N* = 952	Cross-sectional bio-behavioral study, recruited in drug-dealing/consumption locations and brothels (licensed or not)	Substance Use and Dependence	The Alcohol Use Disorders Identification Test (AUDIT)	45.4% used alcohol at the level of harmful or dangerous use.
				Other	The Kessler PD Scale (K10)	The prevalence of Psychological Distress (PD) significantly differed across the three groups (*p* < 0.001). Among Drug Using sex workers, the prevalence of mild PD was 23.4% (95% CI: 20–28), and 32.1% (95% CI: 28–37) had severe/moderate PD. Severe/moderate PD was reported in 19% of female sex workers (95% CI: 17–22).
Teixeira (2017) (77)	Porto, Portugal	*N* = 52 Street-Based Sex Workers	Cross sectional convenience sample, contacted the participants through intermediaries working in five non-governmental organizations (NGOs)	Suicide and Self-Harm	The Suicidal Ideation Questionnaire (SIQ)	The average score on the Suicidal Ideation Questionnaire (SIQ) for participants in the study was 41.71 (SD = 29.93), significantly higher [*t* (25) = 4.51, *p* < 0.01] than the mean score of 23.04 (SD = 25.65) reported for the Portuguese population. Nearly half of the participants (46.1%) scored at or above the threshold for serious suicidal ideation. About 44,2% of the participants reported having made at least one suicide attempt, with 30,4% reporting three or more attempts.
Ulibarri (2009) (62)	Tijuana and Cd. Juarez, Mexico	*N* = 916; Street worker: 54,80% Bar/Cantina-type setting: 36,70% Brothel (casa de citas): 2,60% Call girl/escort: 1,90% Other: 3.20%	Baseline data from intervention study (randomized trial), recruited through street outreach, health clinics, and referrals from other participants	Depression	Depression subscale from the Brief Symptom Inventory (BSI)	The mean score for depression among 908 participants was 1.47, with a standard deviation of 1.03.
Ulibarri (2013) (56)	Tijuana and Cd. Juarez, Mexico	*N* = 624 Sex Workers who reported Injection Drug Use	Mixed methods study with baseline data from intervention study, recruited via targeted sampling techniques	Depression	10-item, short form of the Center for Epidemiologic Studies, Depression Scale (CES-D)	The average score for depression symptoms among the sample was 17.50, with a standard deviation of 7. Furthermore, 86% of the participants (*n* = 538) had scores exceeding the cut of score (a total score of 10) for depression.
Ulibarri (2015) (57)	Tijuana and Cd. Juarez, Mexico	*N* = 230	Cross-sectional study, recruited by targeted sampling	Depression	10-item, short form of the Center for Epidemiologic Studies, Depression Scale (CES-D)	The study examined female sex workers and their steady (non-commercial) partners. Female sex workers had significantly higher average total scores on the CES-D compared to their male partners. The average depression scores were 11.8 (SD = 6.4) for female sex workers and 8.4 (SD = 5.2) for their partners. This difference was statistically significant, with a p-value of less than 0.001.
Vaddiparti (2006) (108)	St. Louis City, Missouri, USA	*N* = 594, *N* = 362 Street-Based Sex Workers, *N* = 232 non sex workers	Baseline data from two interventional studies: (a) a study of heavy drinking women who did not test positive for cocaine, heroin or amphetamines (b) a study of women who tested positive for cocaine, heroin or amphetamines, recruited via street-based outreach program	Substance Use and Dependence	The Substance Abuse Module (SAM)	85% of sex workers reported DSM-IV cocaine dependence, while only 56% of non sex workers reported cocaine dependence (*p* = 0.0001).
Yesuf (2023) (45)	Dire Dawa City, Ethiopia	*N* = 292	Cross-sectional explanatory sequential mixed-method research design, recruited via snowball sampling	Anxiety	The Beck Anxiety Inventory (BAI)	Participants who reported mild anxiety made up 56.5% of the respondents. 66 participants (22.6%) had moderate anxiety, and 61 participants (20.9%) experienced severe anxiety.
				Depression	The Patient Health Questionnaire-9 (PHQ-9)	107 respondents (36.6%) had severe depression, 74 respondents (25.3%) had moderate depression, 54 respondents (18.5%) had mild depression, and 23 respondents (7.9%) had moderately severe depression. 11.6% had no depression.
Zhai (2023) (27)	Huanggang City, China	*N* = 826	Cross-sectional research design with stratified cluster sampling method	Anxiety	The Self-rating Anxiety Scale (SAS)	A total of 356 female sex workers experienced anxiety, resulting in a prevalence rate of 43.1%. Of these, 344 (41.6%) had mild anxiety, 8 (1%) had moderate anxiety, and 4 (0.5%) had severe anxiety.
				Depression	The Self-rating Depression Scale (SDS)	A total of 270 participants were found to have depression, representing a prevalence rate of 32.7%. Among these, 143 (17.3%) were mildly depressed, 65 (7.9%) were moderately depressed, and 62 (7.5%) were severely depressed.
Zhang (2014) (64)	Guangxi, China	*N* = 1,022	Cross-sectional study, The owners and/or managers of sex work venues were contacted first for permission, the outreach health workers then approached female sex workers in the establishments to ask for their participation, street-based sex workers were recruited through direct personal contact or peer referral	Suicide and Self-Harm	Suicide ideation and attempt according to ICD-10 criteria	The prevalence of suicidal behaviors among female sex workers was 9.5% (97 out of 1,022)

**Table 2:** Risk and resilience factors for mental conditions among sex workers.

First Author, Year	Place	Sample size and population	Study design and recruitment strategy	Outcome variable and mental condition	Measurement	Main results
Beksinska (2021) (101)	Kenya	*N* = 1,003, 91.40% worked from a lodge/hotel/rented room	Baseline cross-sectional data from mixed method longitudinal study, recruited from seven specialized clinic services for sex workers	Anxiety, depression	The Patient Health Questionnaire (PHQ-9), the Generalized Anxiety Disorder tool (GAD-7), the Harvard Trauma Questionnaire (HTQ-17)	Women with moderate to severe depression and/or anxiety had a higher prevalence of Adverse Childhood Experiences (ACEs), with 34.2% of these women reporting an ACE score of 9–12, compared to 17.2% of all women. The association between high ACE scores and depression/anxiety was strong, with an adjusted odds ratio (aOR) of 7 (95% CI: 4.36-11.25, *p* < 0.001). Specific ACEs linked to depression/anxiety included experiences of sexual/physical violence and war/community violence during childhood. Older women (>35 years) were more likely to report depression/anxiety compared to younger women (<25 years), with an aOR of 2.08 (95% CI: 1.31-3.30, *p* = 0.007). Socio-economic factors associated with depression/anxiety included missing meals due to lack of food (aOR = 1.63, 95% CI: 1.13–2.36, *p* = 0.009) and engaging in sex work for additional income (aOR = 1.76, 95% CI: 1.25–249, *p* = 0.001). Depression/anxiety was more common among women who had experienced the death of a child (aOR = 1.62, 95% CI: 1.00-2.62, *p* = 0.05) and less common among those with social support (aOR = 0.59, 95% CI: 0.41–0.85, *p* = 0.005). Women with depression/anxiety were more likely to report non-consensual sexual debut (aOR = 1.90, 95% CI: 1.35–2.67, *p* < 0.001) and perceived sex work stigma (aOR = 3.44, 95% CI: 1.77-6.66, *p* < 0.001). There was no significant association with condom use or HIV/STIs. Additionally, these women were more likely to report recent physical and/or sexual violence from a non-intimate partner (aOR = 1.46, 95% CI: 1.00-2.15, *p* = 0.05).
				Alcohol use	The Alcohol, Smoking and Substance Involvement Screening Test (ASSIST)	Among women with any mental health problem (depression/anxiety/PTSD) almost two thirds (63%) also had a harmful alcohol/substance use problem. One in five women (20.5%) reported the co-occurrence of a mental health problem or suicidal thoughts/behaviors and an alcohol/substance use problem.
				PTSD	The Harvard Trauma Questionnaire (HTQ-17), the WHO Adverse Childhood Experiences (ACE) score, the WHO Violence Against Women questionnaire	PTSD was linked to a higher score on the Adverse Childhood Experiences (ACE) scale (scores between 9 and 12 had an adjusted odds ratio (aOR) of 7.19; 95% CI = 3.84–13.45; *p* < 0.001), which included experiences of sexual/physical violence and homelessness during childhood. Women with PTSD were also more likely to experience recent hunger and rely on additional sources of income besides sex work. The prevalence of PTSD was lower among women who had social support (aOR = 0.59; 95% CI = 0.38–0.94; *p* = 0.03) and those who had children (having 1–2 children: aOR = 0.31; 95% CI = 0.14–0.71; *p* = 0.02). Additionally, women with PTSD showed a higher incidence of recent anal sex (7.6% vs. 1.6%; aOR = 11.7; 95% CI = 3.62–37.59; *p* < 0.001) and chlamydia infections (8.4% vs. 5.7%; aOR = 2.46; 95% CI = 1.15–5.27; *p* = 0.02). They were also more likely to have had an abortion or stillbirth (60.2% vs. 44.6%; aOR = 1.6; 95% CI = 1.03–2.49; *p* = 0.04). Women with PTSD reported a higher likelihood of recent sexual and/or physical violence from intimate partners (IPs) (46.4% vs. 30.9%; aOR = 1.68; 95% CI = 1.08–2.63; *p* = 0.02) and non-IPs (72.2% vs. 55%; aOR = 1.72; 95% CI = 1.03–2.88; *p* = 0.04).
Maclin (2023) (23)	Santo Domingo, Dominican Republic	*N* = 211 Cisgender female, *N* = 100 Transgender Female sex workers living with HIV	Cross-sectional surveys, baseline data from intervention study, recruited via non-random sampling led by peer navigators	Anxiety	The anxiety-specific module from the Hospital Anxiety and Depression Scale (HADS-A)	Those in the Sex Work-related Police Harassment class had almost four times greater likelihood of scoring Borderline Abnormal or Abnormal on the HADS-A (aOR = 3.97; 95% CI 1.60, 9.85; *p* < 0.01).
				Depression	The Patient Health Questionnaire-9 (PHQ-9), conducted as interview	Those who experienced police harassment showed almost six times greater likelihood of having moderate to severe depression based on the PHQ-9 (aOR = 5.74; 95% CI 2.33, 14.11; *p* < 0.001).
Beksinska (2022) (101)	Kenya	*N* = 1,003 Female Sex workers	Baseline cross-sectional data from mixed method longitudinal study, recruited from seven specialized clinic services for sex workers	Anxiety	The Generalized Anxiety Disorder (GAD-7) Tool	Among individuals without anxiety, the prevalence of harmful substance use was reported as follows: 26.3% for alcohol, 15.8% for cannabis, and 20.2% for amphetamines. On the other hand, 109 individuals (11%) reported anxiety (GAD-7 score of 10 or higher). In this group, the rates of harmful substance use were significantly higher: 57.6% for alcohol, 25.2% for cannabis, and 31.3% for amphetamines. Therefore anxiety was associated with higher substance use prevalences.
				Depression	The World Health Organization (WHO) Alcohol, Smoking and Substance Involvement Screening Test (ASSIST), the WHO Adverse Childhood Experiences International Questionnaire (ACE-IQ), The Patient Health Questionnaire (PHQ-9)	Quantitative analysis showed that harmful alcohol use was associated with increased odds of depression/anxiety (aOR = 2.36; 95% CI: 1.58–3.52; *p* < 0.001). Women who used cannabis harmfully (aOR = 2.08; 95% CI: 1.32–3.28; *p* = 0.002) or amphetamines (aOR = 1.95; 95% CI: 1.29–2.96; *p* = 0.002) were also more likely to report increased odds of depression/anxiety.
				Alcohol use	The World Health Organization (WHO) Alcohol, Smoking and Substance Involvement Screening Test (ASSIST)	Women with harmful alcohol use were significantly more likely to have a higher ACE score (ACE score 9–12: aOR = 3.66; 95% CI: 2.34–5.72; *p* < 0.001), to have lived on the street (aOR = 1.95; 95% CI: 1.31–2.92; *p* = 0.001), and to have experienced sexual/physical violence (aOR: 3.27; 95% CI: 2.13–5.02; *p* < 0.001) in childhood, compared with women without harmful alcohol use. Women with harmful alcohol use were more likely to report forced sexual debut (aOR = 2.25; 95% CI: 1.62–3.13; *p* < 0.001). However, women who reported harmful alcohol use had significantly lower HIV prevalence (16.8 vs. 28%; aOR = 0.41; 95% CI: 0.27–0.62; *p* < 0.001).
				Substance Use and Dependence	The World Health Organization (WHO) Alcohol, Smoking and Substance Involvement Screening Test (ASSIST)	Women with harmful cannabis use were more likely to report higher levels of ACEs (ACE score 9–12: aOR = 2.99; 95% CI: 1.72–5.21; *p* < 0.001), to have lived on the street, and to have experienced sexual/physical violence in childhood. Women with harmful amphetamine use were more likely to report having lived on the street, but amphetamine use was not associated with higher ACE levels overall. Harmful alcohol and cannabis were more common among younger women <25 compared to older women >35 (alcohol: aOR = 0.67; 95% CI: 0.46–0.99; *p* = 0.05; cannabis: aOR = 0.27; 95% CI: 0.17–0.43; *p*-value <0.001). Moderate/high risk amphetamine use was more common among women in lower Socio-Economic-Status (SES) groups (aOR for highest SES compared to lowest SES: 0.63; 95% CI: 0.42–0.94; p-value: 0.009), as well as being more commonly reported among Muslim women (aOR = 2.39; 95% CI: 1.17–4.87; *p* = 0.0008) and married women (aOR = 1.68; 95% CI: 1.05–2.67; *p* = 0.03). Women with harmful cannabis use were more likely to report selling sex in public places (aOR = 3.26; 95% CI: 1.23–8.65; *p* = 0.03) and more likely to have recently migrated for sex work (aOR = 1.80; 95% CI: 1.20–2.72; *p* = 0.005). Harmful alcohol and cannabis use were associated with increased odds of recent sexual and/or physical violence from non-intimate partners (alcohol: aOR = 1.60; 95% CI: 1v13–2.27; *p* = 0.008; cannabis: aOR = 2.03; 95% CI: 1.31–3.15; *p* = 0.001), as well as of recent arrest.
Zhai (2023) (27)	Huanggang City, China	*N* = 826	Cross-sectional study with stratified cluster sampling method	Anxiety, depression	The Self-rating Depression Scale (SDS), the Self-rating Anxiety Scale (SAS), The Pittsburgh Sleep Quality Index (PSQI), Social Support Rating Scale (SSRS), a six-item scale from the Brief Symptoms Inventory Survey (BSI)	A higher proportion of female sex workers who were married, lived in the county, and had a low education level (junior high school or below) were in the non-anxiety group compared to the anxiety group (*P* < 0.001). Conversely, more female sex workerswith a monthly income exceeding 5,000 RMB and those living alone were in the anxiety group (*P* < 0.05). Regarding lifestyle habits, a greater proportion of female sex workers who smoked, and drank alcohol were in the anxiety group (*p* < 0.001). Additionally, more female sex workers quarantining due to the pandemic were in the anxiety group (*p* < 0.001). Quarantining due to the pandemic and exercising were independent risk factors for anxiety in female sex workers (*p* < 0.05), while living in the county and having a sense of social support were protective factors (*p* < 0.05). Anxiety and depression were found to be positively correlated, with a correlation coefficient of 0.72 (*p* < 0.01). PSQI scores were also positively correlated with anxiety and depression, with correlation coefficients of 0.09 and 0.07, respectively (*p* < 0.05). Social support was negatively correlated with anxiety, depression, and PSQI scores, with correlation coefficients of −0.66, −0,58, and − 0.09, respectively. The mean depression score was 10.91 with a standard deviation of 4.98 (on a scale from 7 to 35). Among sex workers who experienced intimate partner violence, the mean score was 11.46 with a standard deviation of 5.04, while among those who did not experience intimate partner violence, the mean score was 10.26 with a standard deviation of 4.83.
Kelton (2023) (44)	35 U.S. States and Puerto Rico	*N* = 102 Stripper	Cross-sectional study, recruited via snowball sampling	Anxiety	The Generalized Anxiety Disorder Scale-7 (GAD-7)	Higher anxiety symptoms were significantly associated with greater current financial concern and, to a lesser degree, greater reported percent of lost income and lower social capital. Depressive and anxiety symptoms did not differ by minority status with regard to racial identity or sexual orientation.
				Depression	The Stigma Scale Short Form by Harvey, The Intersectional Discrimination Index (InDI), The Generalized Anxiety Disorder Scale-7 (GAD-7), The Patient Health Questionnaire-8 (PHQ-8), The Connor–Davidson Resilience Scale 2-Item (CD-RISC 2)	A regression analysis examining the predictors of depressive symptoms identified financial concerns, discrimination, and social capital as significant factors, explaining 28% of the variance in depressive symptoms. Financial concerns had the most substantial impact, accounting for 11% of the variance, followed by discrimination, which accounted for 4%. Social capital, however, was not a significant predictor of depressive symptoms. The study underscores the impact of COVID-19 on mental health, particularly highlighting the significant role of financial concerns and discrimination in worsening depressive symptoms among the participants.
Yesuf (2023) (45)	Dire Dawa City, Ethiopia	*N* = 292	Cross-sectional explanatory sequential mixed-method research design, recruited via snowball sampling	Anxiety	The Beck Anxiety Inventory (BAI)	Street sex workers (M = 27.5, SD = 9.8) had higher anxiety level than respondents who worked in their home (M = 21.7, SD = 7.9). The overall model implied that 56% of the variations in anxiety were explained by the 8 predictor variables included in the model. The highest contribution in explaining anxiety came from Khat use (*p* < 0.01). That is followed by violence (*p* < 0.01), stigma (*p* < 0.01), tobacco use (*p* < 0.01) and alcohol use (*p* < 0.01). Service years, education and marijuana use were insignificant predictors of anxiety.
				Depression	The Patient Health Questionnaire-9 (PHQ-9)	The model demonstrated that when all predictor variables were considered together, they significantly predicted 23% of the variations in depression (*F* = 10.319, *p* < 0.01, R^2^ = 0.228). The standardized beta coefficient values indicated that the highest contribution to explaining depression came from violence (*β* = 0.298, t = 5,439, *p* < 0.01), followed by alcohol use (*β* = −0.162, t = 3.011, *p* < 0.01), stigma (*β* = 0,160, t = 2,549, *p* < 0.05), and Khat use (*β* = 0,151, t = 2,915, *p* < 0.01). The t-test results of the beta coefficient values showed that tobacco use, marijuana use, service years, and educational status were insignificant predictors of female sex workers’ depression.
Decker (2022) (40)	Mae Sot, Thailand	*N* = 128 Migrant sex workers	Mixed-methods study including a cross-sectional survey, recruited by respondent driven sampling	Anxiety	The Hopkins Symptoms Checklist-25 (HSCL-25)	Anxiety was more prevalent among those who experienced fraud, force or coercion (79,8% vs. 64,7%; AOR: 2,65, 95% CI: 1,68 - 4,13).
Krumrei-Mancuso (2017) (5)	Netherlands	*N* = 88, 29.5% worked in a sex club or brothel, 28.4% from home, 23.8% as escorts, 14.8% from a window, 6.8% outdoors, and 2%in massage salons	Cross-sectional, recruited through personal outreach at each window of 9 red light districts in 6 cities, flyers were mailed to 106 sex clubs/brothels in 60 cities with the request to post them for workers and through direct messages to sex workers	Depression	The 10-item Center for Epidemiologic Studies Depression Scale (CES-D10), The Brief Personal Meaning Profile (PMP-B)	Individuals who engaged in sex work for economic reasons exhibited higher levels of depression, with 53,4% scoring 10 or above on the depression scale. In contrast, those who participated for enjoyment or a combination of financial and pleasurable motives had lower incidences of depressive symptoms, with 7.6% of those engaging for fun or excitement and 14.2% of those involved for both financial reasons and enjoyment showing notable levels of depression. There were no significant differences in levels of depressive symptoms across different work settings. Regression analysis indicated that higher confidence in securing an alternative job was associated with fewer depressive symptoms (b = 0.30, *p* < 0.05).This job confidence explained 7.4% of the variance in depressive symptoms beyond what age and education could account for. No significant differences were found between those who experienced violence in sex work and those who did not regarding depressive symptoms. The quality of life questionnaire revealed that focusing on achievement and perceiving fair treatment in life were linked to fewer depressive symptoms (b = 0.56, *p* < 0.05 and b = 0.45, *p* < 0.01). Conversely, a tendency towards self-transcendence was associated with increased depressive symptoms (b = 0.37, *p* < 0.05).
				Ptsd	The Post-Traumatic Stress Disorder Check List – Civilian Version (PCL-C)	For Post-traumatic stress, the means and standard deviations were (according to working place): Window (M = 0.66, SD = 0.79), Brothel (M = 0.60, SD = 0.74), Escort (M = 0.90, SD = 0.85), Home (M = 0.53, SD = 0.57), Combination including outdoors (M = 1.97, SD = 1.31), and Combination excluding outdoors (M = 0.66, SD = 0.69). The F statistic was 3,13 with a significance of *p* < 0.05, indicating a significant difference in post-traumatic stress levels across some settings, particularly between the combination including outdoors and other settings. Intentions about how long to remain in prostitution were not predictive of levels of post-traumatic stress (b = −0.21, *p* = 0.32). However, a greater desire to leave prostitution was associated with higher levels of post-traumatic stress (b = 0.51, *p* < 0.001). Desire to leave prostitution accounted for 26% of the variance in post-traumatic stress. Greater confidence in finding an alternative job was also associated with less post-traumatic stress (b = −0.34, *p* < 0.05) after controlling for age. Confidence in finding an alternative job accounted for 10.2% of the variance in post-traumatic stress beyond age.
Carlson (2017) (61)	Ulaanbaatar, Mongolia	*N* = 222	Baseline data from intervention study for sex workers with alcohol use disorder, no recruitment strategy described	Depression	The six-item depression subscale of the Brief Symptom Inventory (BSI), the Multidimensional Scale of Perceived Social Support, self-designed stigma scale ranging from 0 to 24, the Revised Conflict Tactics Scale (CTS2), the Alcohol Use Disorders Identification Test (AUDIT)	Linear regression analysis revealed several significant predictors of depressive symptoms. There was a negative correlation between social support and depressive symptoms (*β* = −0.27, *p* < 0.0001), indicating that higher social support is associated with fewer depressive symptoms. Stigma associated with engaging in sex work was a significant predictor of depressive symptoms (*β* = 0.12, *p* = 0.05). Experiencing sexual violence from a paying partner was strongly linked to higher depressive symptoms (*β* = 0.26, *p* < 0.0001), as was elevated harmful alcohol use (*β* = 0.27, *p* < 0.0001). However, factors such as age, having a trusted partner, number of dependents, possessing a secondary degree or higher, duration of engaging in sex work, total number of sex acts, proportion of unprotected sex acts, and history of physical violence from a paying partner did not show a significant relationship with depressive symptoms.
MacLean (2018) (55)	Lilongwe, Malawi	*N* = 200	Cross-sectional study, venue-based sampling at 23 establishments through outreach team	Depression	The Patient Health Questionnaire (PHQ-9)	In the bivariable analysis, female sex workers who had completed primary education were slightly more likely to experience probable depression, with a prevalence ratio (PR) of 1,90 (95% confidence interval [CI]: 0,97 – 6,40).
				Ptsd	The Post-Traumatic Stress Disorder Check List – Civilian Version (PCL-C)	Female sex workers with ≥20 clients per week were 2.4 (95% CI: 1–11.8) times as likely to have probable PTSD. The effect increased slightly in the multivariable model (aOR 3.3, 95% CI: 1–11.2).
Pandiyan (2018) (66)	Bangalore, Karnataka, India	*N* = 246	Cross-sectional study, recruited from a psychiatry	Depression	The Beck’s Depression inventory 2 (BDI-II)	Sex workers with substance use had higher prevalence of depression. There was a significant correlation between alcohol use severity and depression.
Coetzee (2018) (67)	Soweto, South Africa	*N* = 508	Cross-sectional study, recruited through respondent driven sampling	Depression	The 20-item Center for Epidemiologic Studies Depression Scale (CES-D), the Alcohol Use Disorders Identification Test (AUDIT-C), Internal stigma were assessed using an adaptation of the People Living with HIV Stigma Index, Self-esteem was assessed using the 10-item Rosenberg Self-esteem Scale, the WHO violence against women questionnaire	Among the depressed female sex workers, 89.9% reported having been pregnant, and 20.1% had experienced the loss of a child. Additionally, 60.1% of the depressed participants frequently faced situations where they went without eating. The occurrence of violence varied, with 12.9% reporting no exposure to violence, 24.2% experiencing one form of violence, 29.5% encountering two forms of violence, and 33.5% facing three or more types of violence. The median score for internalized stigma was 14 (interquartile range 13 to 16), and 54% of those with depression participated in severe binge drinking. Among those showing symptoms of depression, the median self-esteem score was 11 (interquartile range 9 to 13).
				Ptsd	The Posttraumatic Stress Disorder-8 (PTSD-8) Inventory	The only notable distinction observed was in the sleeping arrangements over the past week among different PTSD groups. Specifically, 36.9% of those affected by PTSD had spent nights with family members, while 36.6% stayed in locations associated with sex work. This contrasts with 55.1% who slept with family or in places not related to sex work. Among female sex workers impacted by PTSD, the distribution of co-occurring violence experiences was as follows: 11.1% reported none, 25.8% encountered one type, 29.4% experienced two types, and 33.7% faced three or more types. The median score for internalized stigma was 14, within an interquartile range of 13 to 16. Furthermore, severe binge drinking was observed in 57.6% of the female sex workers who were suffering from PTSD.
				Other	The 20-item Center for Epidemiologic Studies Depression Scale (CES-D), the Alcohol Use Disorders Identification Test (AUDIT-C), Internal stigma were assessed using an adaptation of the People Living with HIV Stigma Index, Self-esteem was assessed using the 10-item Rosenberg Self-esteem Scale, the WHO violence against women questionnaire, the childhood trauma questionnaire (CTQ)	The multinomial logistic regression compared no mental health concerns with either depression or PTSD independently or comorbidity. The weighted analysis shows that an increasing age of first selling sex increased the likelihood of having one of the two mental health concerns (RRR 1.09, 95% CI: 1.04 –1.14, *p* = 0.001). Experiencing three or more kinds of violence increased the likelihood of comorbid conditions by four times (RRR 4.11, 95% CI: 1.52–11.12, *p* = 0.005) and also affected the likelihood of an independent condition by three times (RRR 300, 95% CI 1.22–7.39, *p* = 0.017). The internalization of stigma increased the likelihood of having an independent mental health condition (RRR 1.25, 95% CI 1.10–1.42, *p* = 0.001), while higher self-esteem reduced the likelihood of both independent conditions (RRR 0.88, 95% CI 0.80–0.97, *p* = 0.002) and comorbid conditions (RRR 0.85, 95% CI 0.76–0.95, *p* = 0.001).
Abelson (2019) (25)	Yaoundé, Douala, Bertoua, Kribi and Bamenda, Cameroon	*N* = 2,165	Cross-sectional study (baseline data from intervention study), recruited through respondent-driven sampling	Depression	The Patient Health Questionnaire (PHQ-9)	As the level of depression escalated, the percentage of individuals reporting experiences of sexual violence rose. Specifically, among participants without depression, 24.9% (267 out of 1,071) disclosed incidents of sexual violence. In contrast, 56.1% (32 out of 57) of those with severe depression reported having experienced sexual violence, indicating a significant increase (*p* < 0.01).
Murnan (2018) (68)	Columbus, Ohio, USA	*N* = 183 Sex Workers who were Mothers with Substance Use Disorder	Randomized Control Trial, recruited from a large substance use treatment facility	Depression	The Beck Depression Inventory II (BDI-II)	Maternal race significantly influenced depression outcomes negatively (b = −7.81, s.e. = 3.56, t = −2.20, *p* = 0.03), with African American mothers reporting higher levels of depressive symptoms compared to mothers of other races, primarily white.
Stockton (2020) (63)	Nairobi, Kitui, Busia, and Homa Bay, Kenya	*N* = 497 Female sex workers, *N* = 232 Male sex workers	Cross-sectional survey, recruited via snowball sampling	Depression	The Patient Health Questionnaire–9 (PHQ-9), the validated Sex Work Experienced Stigma Scale (SWESS)	Increasing levels of experienced stigma were associated with a higher predicted prevalence of depression: aOR 0.15 (95% CI 0.11–0.18). Additionally, rising internalized stigma was independently linked to higher experienced stigma and depression, accounting for 25.5% of the shared variance between experienced stigma and depression after adjusting for confounders.
Kim (2020) (58)	Chicago, Illinos, USA	*N* = 400, *N* = 181 sex workers, *N* = 92 Care/Service work only *N* = 68 engaged in both	Cross-sectional study, recruited from jail	Depression	The Center for Epidemiologic Studies Depression Scale (CESD), the Medical Outcomes Study (MOS) Social Support Survey	Black participants had a higher odds ratio (OR) for clinical depression (1, 17). Higher social support was significantly associated with lower odds of clinical depression (OR = 0.97, *p* < 0.01). Having less than a high school education was not associated with depression. First-time incarceration was associated with significantly higher odds of clinical depression (OR = 2.66, *p* < 0.05).
Ouma (2021) (69)	Northern Uganda	*N* = 300	Cross-sectional study, recruited through telephone from the database of The AIDS Support Organization (TASO)	Depression	The Mini-International Neuropsychiatric Interview (M.I.N.I.)	Life stress (adjusted OR = 10.8, 95% CI: 5.67–20.57), living with HIV (adjusted OR = 2.25, 95% CI: 1.25–4.05), verbal abuse (adjusted OR = 2.27, 95% CI: 1.27–4.08), and older age (adjusted OR = 106, 95% CI: 1.01–1.12) were all positively associated with major depression. Conversely, providing sexual services from clients’ homes (adjusted OR = 0.50, 95% CI: 0.25–0.97), using a non-barrier modern family planning method (adjusted OR = 0.44, 95% CI: 0.24–0.82), and daily alcohol intake (adjusted OR = 0.50, 95% CI: 0.28–0.88) were negatively associated with major depression.
Bhardwaj (2023) (51)	eThekwini, South Africa	*N* = 1,384	Baseline data from adaptive intervention study, recruited by the peer case managers at known sex work venues	Depression	The Patient Health Questionnaire (PHQ-9)	In the age-adjusted multivariate model, the prevalence of depression was associated with experiencing physical violence more than five times in the past six months (aOR = 1.38, 95% CI: 1.07 - 1.80) compared to experiencing no physical violence in the same period. Additionally, having any lifetime experience of sexual violence (aOR = 1.47, 95% CI: 1.07–1.80) and using illicit drugs in the past month (aOR = 1.23, 95% CI: 1.04–1.48) were also associated with depression. Higher levels of internalized stigma were similarly linked to depression (aOR = 1.11, 95% CI: 1.04 – 1.18).
Hendrickson (2023) (71)	Baltimore, Maryland, USA	*N* = 370 Street-Based Sex Workers	Baseline, 6-month, and 12-month follow-up data from interventional study, recruited using time–location sampling	Depression	The Patient Health Questionnaire (PHQ-9), conducted as interview	Elevated levels of depressive symptoms, were observed in 168 participants (46%), with low mobility accounting for 72 (40.4%) of these cases and mobile sex workers making up 96 (51.3%), showing a statistically significant difference with a p-value of 0.037.
				Ptsd	The PTSD Checklist for DSM-5 (PCL-5)	Those with scores suggesting more severe PTSD symptoms had a significantly increased risk of experiencing physical and sexual violence, respectively, compared to those with lower PCL-5 scores (physical aRR: 1.45, 95% CI: 1.09–1.92, *p* < 0.05; sexual aRR: 1.47, 95% CI: 1.04–2.09, *p* < 0.05).
Sherwood (2024) (50)	Pattaya, Thailand	*N* = 232 Benue-Based Sex Workers	Secondary data analysis from a quasi-experimental study, recruited via venue-based sampling from two sex work zones/hotspots.	Depression	The two-question Patient Health Questionnaire (PHQ-2)	Women who experienced client violence at baseline were more likely to have depressive symptoms (19% vs.7.5%, *p* < 0.05), female sex workers who experienced sex with clients while inebriated in the past 3 months at follow up (n = 118, 51%) were more likely to have depressive symptoms (14.4% vs. 6.1%, *p* < 0.05).
Nabayinda (2023) (29)	Southern Uganda	*N* = 542	baseline data from interventional study, recruited from 19 HIV hot spots across four districts in Southern Uganda	Depression	A six-item scale from the Brief Symptoms Inventory Survey (48), conducted as interview	The mean depression score was 10.91 with a standard deviation of 4.98 (on a scale from 7 to 35). Among sex workers who experienced intimate partner violence, the mean score was 11.46 with a standard deviation of 5.04, while among those who did not experience intimate partner violence, the mean score was 10.26 with a standard deviation of 4.83.
Tomko (2023) (90)	Baltimore, Mayland, USA	*N* = 367 (mostly) Street-Based Sex Workers	The study’s recruitment process was guided by a prior 12-month longitudinal cohort study of Female Sex Workers in Baltimore City, which utilized targeted sampling techniques, recruitment was done in known hot spots	Depression	The Internalized Sex Work Stigma Scale (ISWSS), the Patient Health Questionnaire-9 (PHQ-9)	Depression was significantly correlated with the ISWSS [*r* (359) = 0.44, *p* < 0.001], worthlessness [*r* (359) = 0.51, *p* < 0.001], guilt/shame [*r* (359) = 0.38, *p* < 0.001], and sex work illegitimacy [*r* (359) = −0.17, *p* = 0.001].
				PTSD	The PTSD Checklist for DSM-5 (PCL-5), the Alcohol Use Disorders Identification Test (AUDIT-C)	Women with high PCL-5 scores were more than twice as likely to have used benzodiazepines recently (risk ratio [RR] = 2.78, 95% confidence interval [CI] = 1.62–4.76) compared to women with low scores; there were no significant differences between medium versus low scores. Women with high Intrusive Thoughts scores were significantly more likely to have recently used benzodiazepines non-medically compared to women with low scores (aRR = 1.77, 95% CI = 1.12–2.79). Similarly, women with high Avoiding Reminders scores were more likely than women with low scores to have used benzodiazepines non-medically (aRR = 2.30, 95% CI = 1.49–3.54). Finally, women with medium (aRR = 1.82, 95% CI = 1.07–3.08) and high (aRR = 1.80, 95% CI = 1.07–3.04) Negative Alterations in Arousal/Reactivity scores were more likely to have recently used benzodiazepines non-medically compared to women with low scores.
Grudzen (2011) (53)	California, USA	*N* = 134 Adult Film Performer *N* = 1,773 women of similar ages	Cross-sectional structured online survey, recruited via Email	Depression	The Patient Health Questionnaire–8 (PHQ-8)	After adjusting for demographic characteristics, general health risks, and behaviors, significant predictors of current depression included general medical health, poverty, domestic violence, forced sex as an adult, and growing up in a household receiving welfare (*p* < 0.05). Notably, being an adult film performer, experiencing foster care as a child, and forced sex before the age of 18 did not emerge as significant predictors of depression. The analysis highlighted that forced sex in childhood or adolescence became a significant predictor of depression (*p* < 0.05), while adult film performer status did not predict current depression after controlling for the fore mentioned covariates.
Roxburgh (2006) (9)	Sydney, Australia	*N* = 72 Street-Based Sex Workers	Cross-sectional study, recruited through agencies	Depression	The Beck Depression Inventory-II (BDI-II)	National Aboriginal and Torres Strait Islander (A&TSI) women reported significantly higher levels of depression (BDI mean of 36.5) compared to non-A&TSI women (6, 26) (t = 2.8, df = 68 *p* = 0.007).
				Ptsd	The National Mental Health and WellBeing (NHMWB) version of the Composite International Diagnostic Interview (CIDI)	Women reporting current PTSD were nearly four times more likely to have ever experienced adult sexual assault than women without current PTSD (82% vs. 53%, respectively; *χ*^2^ = 4.18, OR 3.98, 95% CI: 1.2 - 13.5). They also experienced a significantly greater number of traumas (median of 7 traumas) compared to those without current PTSD (median of 5 traumas) (Mann Whitney U = 329, df = 70, *p* < 0.01). Additionally, women with current PTSD were nearly four times more likely to report being seriously neglected as a child (59%) than women without current PTSD (28%) (*χ*^2^ = 5.04, OR 3.7, 95% CI 1.2–10.6). There were no differences in the proportions reporting child sexual abuse (82% among those with current PTSD and 72% among those without current PTSD) or physical assault at work (77% each). Similarly, there was no difference between the groups in the median age of first sexual assault (13 for those with current PTSD and 14 for those without current PTSD).
Roxburgh (2008) (113)	Sydney, Australia	*N* = 72 Street-Based Sex Workers	Cross-sectional study, recruited through agencies	Substance Use and Dependence	Severity of Dependence Scale (SDS)	Women who had left home before age 16 were 4.2 times more likely to report being cocaine dependent than those who had not (*χ*^2^ = 6.85, CI 95% 1.4 to 13.05). Female sex workers who were cocaine dependent commenced injecting drug use at a significantly younger age than those who were not (*p* < 0.01).There were no differences between those who were cocaine dependent and those who were not on mental health variables such as depression and current PTSD; however, women who were dependent were 4.7 times more likely to report having been raped at work than those who were not dependent (*χ*^2^ = 4.7, CI 95% 13 to 11.2).
Suresh (2009) (85)	Chennai, India	*N* = 57 Street-Based Sex Workers	Cross-sectional study, recruited randomly through an agency	Depression	The Center for Epidemiologic Studies Depression scale (CES-D), partner violence scales were adapted from the WHO’s Multi-Country Study on Women’s Health and Domestic Violence	Violence and depression have a moderate correlation with r = 0.33 (significant at *α* ≤ 0.01). Respondents who experienced higher levels of violence at work and at home had higher measures of depression (*χ*^2^ = 7.27, *α* ≤ 0.01). Those who experienced childhood neglect, in addition to neglect by their husbands and violence at work, also had higher measures of depression (*χ*^2^ = 7.54, α ≤ 0.05). The findings suggest that compared to sex workers with high depression scores, women with low depression scores experienced less childhood trauma, less neglect by husbands, and less violence at work.
				Suicide and Self-Harm	The 20-item Center for Epidemiologic Studies Depression Scale (CES-D)	Severe depression in the sample is 166 times more likely to explain suicidal ideation (statistically significant at α ≤ 0.05 level). Suicidal ideation and alcohol use in this study had a weak correlation (r = 0.13).
				Ptsd	The PTSD Checklist for DSM-5 (PCL-5)	The mean PTSD and depression scores are higher for those who experience violence in their work compared to those who do not. The mean depression and PTSD scores for individuals who believe they experienced severe violence versus those who consider the violence they faced to be less severe. Sex workers who encountered more violence while in the profession have higher mean scores of depression and PTSD. These respondents noted that the sex work itself is not depressing or stressful, but the violence they encounter is most depressing and painful for them, especially when it is experienced more frequently.
Ulibarri (2009) (62)	Tijuana and Cd. Juarez, Mexico	*N* = 916 female sex workers, Street worker: 54.80% Bar/Cantina-type setting: 36.70% Brothel (casa de citas): 2.60% Call girl/escort: 1.90% Other: 3.20%	baseline data from intervention study (randomized trial), recruited through street outreach, health clinics, and referrals from other participants	Depression	The Brief Symptom Inventory (BSI), the Addiction Severity Index (ASI). Social support was assessed using seven items measuring availability of emotional support (Pearlin, Mullan, Semple, & Skaff, 1990)	Age was significantly and negatively related to current depression (r = −0.09, *p* < 0.01), indicating that older individuals had lower mean scores of depression symptoms. Similarly, the number of dependents was significantly and negatively related to current depression symptoms (r = −0.088, *p* < 0.01), meaning that women with more financial dependents had fewer depressive symptoms. Women with histories of abuse in their lifetime were more likely to exhibit more symptoms of depression. Social support was significantly and negatively related to current symptoms of distress, with women having higher levels of social support reporting fewer depression symptoms. Social support accounted for 5% of the variance, while abuse accounted for 9% of the variance in depressive symptoms. Women who had experienced abuse reported more symptoms of depression, and those with less social support also reported more symptoms of depression. Examination of the beta weights for each of the abuse items revealed that the association between a history of sexual abuse and current symptoms of depression was almost twice as strong as for the other two dimensions of abuse.
Hong (2013) (54)	Guangxi, China	*N* = 1,022	Cross-sectional survey, recruited from 60 establishments that represented all known commercial sex venues	Depression	The Center for Epidemiologic Studies Depression scale (CES-D), Partner violence scales were adapted from the WHO’s Multi-Country Study on Women’s Health and Domestic Violence	The mean CES-D score among female sex workers who had experienced physical violence (PV) from a stable partner was 17.9 (SD = 9.2), compared to 14.8 (SD = 9) in their counterparts. Women who reported violence from clients also had higher CES-D scores (mean = 18) than their counterparts (mean = 16.3). After controlling for potential confounders, PV from a stable partner remained significantly associated with depression (aOR = 2). PV from clients was also significantly associated with depression (aOR = 1.76). Another risk factor for depression was working in lower-end commercial sex venues.
Ulibarri (2013) (56)	Tijuana and Cd. Juarez, Mexico	*N* = 624 female sex workers who reported Injection Drug Use (IDU)	Mixed-Methods study with baseline data from intervention study, recruited via targeted sampling techniques	Depression	10-item, short form of the Center for Epidemiologic Studies, Depression Scale (CES-D-10)	Education level was the only demographic factor linked to depression symptoms (*β* = −0.21, *p* = 0.03, 95% CI: −0.41, −0.02). Female sex workers with IDU with higher depression symptoms were more likely to have histories of forced sex (*β* = 2.32, *p* < 0.001, 95% CI: 1.23, 3.41), physical abuse (*β* = 1.66, *p* < 0.001, 95% CI: 0.55–276), physical abuse by a client (*β* = 1.90, *p* = 0.01, 95% CI: 0.55 – 3.25), and forced sex related to sex work (*β* = 265, *p* < 0.001, 95% CI: 1.39 - 3.90). Drug abuse factors did not show a significant relationship with depression symptoms. Female sex workers with IDU who had experienced forced sex had an increased likelihood of current depression symptoms (*β* = 1.49, *p* = 0.02, 95% CI: 0.22 – 2.76), as did female sex workers with IDUs who had undergone forced sex in sex work contexts (*β* = 1.76, *p* = 0.03, 95% CI: 0.21 – 3.32).
Zhang (2014) (64)	Beihai and Guilin, Guangxi Zhuang Autonomous Region (Guangxi) of China	*N* = 1,022	Cross-sectional study, recruited from different venues	Depression	The Center for Epidemiologic Studies Depression Scale (CES-D), the Alcohol Use Disorders Identification Test (AUDIT), measures of partner violence included violence perpetrated by stable partners and violence perpetrated by clients. Both scales were adapted from the World Health Organization (WHO)‘s Women’s Health and Life Experience Questionnaire	Factors positively and significantly associated with depression included alcohol use (OR = 1.05, 95% CI = 1.03, 1.07), drug use (OR = 1.90, 95% CI = 1.37 – 2.64), partner violence (OR = 1.75, 95% CI = 1.35–2.26), home residency (OR = 1.30, 95% CI = 1.01 – 1.67), levels of work venues (OR = 1.16, 95% CI = 1–1.35), and whether the venues serve alcohol (OR = 1.32, 95% CI = 1.03–1.69). Women who used alcohol were more likely to have elevated depressive symptoms (aOR = 1.06, 95% CI = 1.04 - 1.08).
				Suicide and Self-Harm	The Center for Epidemiologic Studies Depression Scale (CES-D), the Alcohol Use Disorders Identification Test (AUDIT), measures of partner violence included violence perpetrated by stable partners and violence perpetrated by clients. Both scales were adapted from the World Health Organization (WHO)‘s Women’s Health and Life Experience Questionnaire	Variables that were significantly and positively associated with suicidal behaviors in the bivariate analyses were alcohol use (OR = 1.06, 95% CI = 1.04 – 1.09), drug use (OR = 3.78, 95% CI = 243 - 5.87), and partner violence (OR = 2.89, 95% CI = 1.70 - 4.90). Age was negatively and significantly associated with suicidal behaviors (OR = 0.95, 95% CI = 0.91 – 0.99).
				Other	Center for Epidemiologic Studies Depression Scale (CES-D), the UCLA Loneliness scale, the Beck Hopefulness Scale, the WHO’s Women’s Health and Life Experience Questionnaire	Female sex workers who reported better relationships with gatekeepers experienced lower levels of depression (*β* = −0.28, 95% CI = −0.46 to −0.10), loneliness (*β* = −0.27, 95% CI = −0.41 to −0.13), and perceived stigma (*β* = −0.3, 95% CI = −0.44 to −0.22), and had a more positive outlook on the future (*β* = 0.10, 95% CI = 0.04 to 0.16) compared to those with poorer relationships with gatekeepers. Even when factors such as alcohol use, drug use, and partner violence were included in the regression analysis, the relationship between female sex workers and gatekeepers still significantly influenced loneliness (*β* = −0.29, 95% CI = −0.47 to −0.12), perceived stigma (*β* = −0.25, 95% CI = −0.44 to −0.07), and suicidal thoughts or attempts (adjusted odds ratio = 0.9, 95% CI = 0.83 to 0.99), and remained positively related to hopefulness (*β* = 0.09, 95% CI = 0.01 to 0.16).
Zhang (2014) (76)	Beihai and Guilin, Guangxi Zhuang Autonomous Region (Guangxi) of China	*N* = 1,022	Cross-sectional study, recruited from different venues	Depression	Center for Epidemiologic Studies Depression Scale (CES-D), the UCLA Loneliness scale, the Beck Hopefulness Scale, the WHO’s Women’s Health and Life Experience Questionnaire	Compared with women who had a worse relationship with gatekeepers, female sex workers who had better gatekeeper relationship had less depression (*β* = −028, 95% CI = −0.46,− 0.10), women who had alcohol and drug use problems, ever experienced partner violence and worked longer in the city were at a higher risk of depression problems (*p* < 0.05).
Patel (2015) (59)	Andhra Pradesh, Southern India	*N* = 1986	Cross-sectional survey, probability sampling (for public venues) and conventional cluster sampling (for nonpublic venues) methods were used	Depression	The Patient Health Questionnaire-2 (PHQ-2)	Among female sex workers who reported having major depression, over half were currently married (53%) and had formal education (63%); 62% were engaged in public places/street-based solicitation; and 69% cited poverty-related circumstances as their main reason for entering the sex trade. More than one-third of these female sex workers (39%) were widowed, deserted, separated, or divorced. Most female sex workers with major depression primarily engaged in sex work in urban or semi-urban areas (83%). The likelihood of experiencing major depression was significantly higher among sex workers with no individual autonomy (50% vs. 20%, AOR = 4.5, 95% CI = 3.2-5.9) and those who had consumed alcohol in the past 30 days (52% vs. 28%, AOR = 2.7, 95% CI = 2–3). The probability of reporting major depression was also significantly higher among female sex workers who had experienced arrest by the police (55% vs. 36%, AOR = 2.1, 95% CI = 1.2-2.6), lived with non-family members (52% vs. 38%, AOR = 2, 95% CI = 1.3-2.9), were mobile for sex work within or outside the district (49% vs. 33%, AOR = 1.9, 95% CI = 1.4-2.4), reported inconsistent condom use with regular (51% vs. 33%, AOR = 1.9, 95% CI = 1.5-2.5) and occasional clients (50% vs. 35%, AOR = 1.6, 95% CI = 1.2-2.2), and knew their HIV positive status or did not want to report their HIV status (46% vs. 36%, AOR = 1.6, 95% CI = 1.2-2.1) compared to others. Female sex workers who had experienced any form of physical violence in the past 6 months (46% vs. 38%, AOR = 1.5, 95% CI = 1.1-2.1) and were under financial debt (41% vs. 31%, AOR = 1, 95% CI = 1.1-2.2) had a 50% higher chance of reporting major depression than those who were not. Those who had experienced any STI symptoms in the past 6 months (46% vs. 38%, AOR = 1.4, 95% CI = 0.9–2) were also more likely to report major depression compared to those who did not experience such symptoms. The results for those who were in financial debt and had experienced physical violence in the past 6 months were not significant.
Patel (2016) (60)	Andhra Pradesh, Southern India	*N* = 2,400	Cross-sectional survey, probability sampling (for public venues) and conventional cluster sampling (for nonpublic venues) methods were used	Depression	The Patient Health Questionnaire-2 (PHQ-2)	The results indicate that experiences of violence and mobility for sex work were independently associated with major depressive symptoms. The likelihood of having major depressive symptoms was three times higher among female sex workers who were mobile outside the district (48% vs. 22%, AOR 2.8, 95% CI 2.2–3.6) and 40% higher among those who were mobile within the district of residence (30% vs. 23%, AOR 1.4, 95% CI: 1.2 to 1.8) compared to those who were not mobile for sex work. Furthermore, female sex workers who were mobile outside the district and reported violence were six times more likely to be at risk for major depressive symptoms (62% vs. 19%, AOR 6.1, 95% CI 4.4–8.6) than female sex workers who were not mobile and did not experience violence. Similarly, female sex workers who were mobile within the district and reported violence were three times more likely to be at risk for major depressive symptoms (43% vs. 19%, AOR 3, 95% CI: 2.2 to 4.2) than those who were not mobile and did not experience violence. The odds of screening positive for major depression were seven times higher among female sex workers who reported any violence perpetrated by the police (73% vs. 23%, AOR 7.4, 95% CI: 4.5 to 13) and five times higher among female sex workers reporting violence perpetrated by a broker, pimp, or goons (62% vs. 23%, AOR 5, 95% CI: 3.4 to 7.6) compared to those who had not experienced any violence.
Zhang (2017) (80)	Beihai and Guilin, Guangxi Zhuang Autonomous Region (Guangxi) of China	*N* = 971	Cross-sectional study, recruited from different venues	Depression	Center for Epidemiologic Studies Depression Scale (CES-D), the UCLA Loneliness scale, the Beck Hopefulness Scale, the WHO’s Women’s Health and Life Experience Questionnaire	Female sex workers who experienced a higher level of violence from stable partners were more likely to report depressive symptoms (*β* = 0.193, 95% CI = 0.26–0.58). The overall scale of violence from clients was also significantly associated with depressive symptoms (*β* = 0.19, 95% CI = 0.31–0.64). The subscale of physical violence from stable partners was significantly associated with depressive symptoms (*β* = 0.136, 95% CI = 0.15–0.5). The subscale of psychological abuse from stable partners was significantly associated with depressive symptoms (*β* = 0.21, 95% CI = 0.21–0.43). Additionally, a stigma subscale, the discredit subscale, was significantly associated with depressive symptoms (*β* = 0.25, 95% CI = 0.14–0.24).
				Suicide and Self-Harm	Center for Epidemiologic Studies Depression Scale (CES-D), the UCLA Loneliness scale, the Beck Hopefulness Scale, the WHO’s Women’s Health and Life Experience Questionnaire	Female sex workers who experienced a higher level of violence from stable partners were more likely to report suicide ideation/attempts (*β* = 0.112, 95% CI = 0.06–0.29), adjusting for education and drug use. The overall scale of violence from clients was also significantly associated with suicide *β* = 012, 95% CI = 0.09–0.33. The subscale of psychological abuse from stable partners was significantly associated with suicide (*β* = 0.14, 95% CI = 0.07–0.22). Female sex workers who experienced a higher level of stigma were more likely to report suicide (*β* = 0.095, 95% CI = 0.02–0.10).
				Alcohol use	Alcohol Use Disorders Identification Test Consumption AUDIT	The mean AUDIT score among the 673 participating female sex workers was 8.4, with a standard deviation of 6.9. Female sex workers with both first and second-degree relatives who drank alcohol had the highest AUDIT scores, indicating a significant correlation (*p* < 0.05). Furthermore, there was a notable trend showing that AUDIT scores and the frequency of intoxication increased with the closeness of family members who consumed alcohol (p for trend <0.05). Women with a higher composite score of Genetic Predisposition to Drinking (GPD) were at a higher risk for alcohol-related problems than their peers. GPD’s association with higher AUDIT scores was consistent regardless of whether the female sex workers underwent regular health or gynecological examinations. This correlation also extended to the number of drinking days per week among women, irrespective of their gynecological check-up status. Female sex workers facing mental health challenges and those with limited access to healthcare were more inclined to report issues with alcohol use.
Chen (2017) (70)	Guangxi, Chongqing, Sichuan, and Xinjiang, China	*N* = 457	Cross-sectional study, recruited from different venues	Depression	Propensity to Trust Survey, Interpersonal Needs Questionnaire, Center for Epidemiological Studies Depression Scale (CES-D)	Age was positively associated with depression scores, with minority groups having higher depression scores than Han Chinese. Additionally, those with children experienced higher depression scores than those without children. The associations between depressive symptoms and other demographic variables were not significant.
Jung (2008) (87)	Korea	*N* = 113 Former Sex Workers *N* = 81 Activists *N* = 111 Control Group	Cross-sectional study, recruited from 18 shelters	Depression	The Stress Response Inventory (SRI)	Sleep troubles were positively correlated to depression (r = 0.58, *p* < 0.001).
				Ptsd	The Davidson Trauma Scale (DTS), the modified Impact of Event Scale-Revised (IES-R), the Stress Response Inventory (SRI)	The length of time spent in prostitution (r = 0.22, *p* = 0.047) and the age of the individuals (r = 0.21, *p* = 0.05) were both found to positively correlate with the severity of PTSD symptoms. Additionally, a significant positive relationship was observed between the severity of PTSD symptoms, and both smoking (r = 0.32, *p* = 0.002) and alcohol consumption issues (r = 0.27, *p* = 0.01). The frequency of PTSD symptoms also demonstrated significant positive correlations with smoking (r = 0.20, *p* = 0.047) and alcohol problems (r = 0.22, *p* = 0.032). Furthermore, sleep disturbances were positively linked to both the frequency (r = 0.45, *p* < 0.001) and severity of PTSD symptoms (r = 0.52, *p* < 0.001). The duration of residence in shelters was negatively correlated to the frequency (r = −0.33, *p* = 0.003) and severity of PTSD symptoms (r = −0.38, *p* = 0.001).
Rossouw (2021) (52)	Nelson Mandela Bay Municipality, South Africa	*N* = 410	Cross-sectional study, recruited via respondent driven sampling	Depression	The Patient Health Questionnaire-9 (PHQ-9)	Participants living with non-family members such as friends, boyfriends, or colleagues (adjusted prevalence ratio 1.38, 95% CI: 1–1.91), as well as those living alone (aPR 1.82, 95% CI: 1.15–2.90), had a higher likelihood of depression compared to those residing with their family (children, husband, or other relatives). Homelessness emerged as a strong indicator of depression (aPR: 2.16, 95% CI: 1.38–3.40). Participants who engaged in sex work due to food insecurity were more than twice as likely to experience depression (aPR: 2.19, 95% CI: 1.42–3.38) compared to those who did not mention food insecurity as a reason. Stigma and police harassment were linked to depression. Individuals experiencing high levels of perceived healthcare stigma were more prone to depression (aPR: 1.35, 95% CI: 0.94–1.93), though this was significant only before adjusting for other factors. Depression was also associated with a lower likelihood of disclosing one’s occupation to healthcare providers (aPR: 0.61, 95% CI: 0.42–0.89). High levels of police harassment (aPR 1.24, 95% CI: 0.85–1.81) and social stigma (aPR: 1.28, 95% CI 0.79–2.07) were observed to increase the likelihood of depression, though these associations were not statistically significant. Sexual violence was significantly associated with depression, while the association with physical violence was significant only in the unadjusted analysis. Participants who experienced rape both as minors and within the last 12 months were 70% more likely to be depressed (aPR: 1.73, 95% CI: 1.01–2.94) compared to those who had never been raped. The relationship between depression and biobehavioral risk factors of HIV showed that neither condom use, HIV status, nor ART status were significantly associated with depression. However, a positive syphilis test (aPR: 1.46, 95% CI: 1.02–2.09) and self-reported STIs in the last 12 months (aPR 1.78, 95% CI: 1.29–2.46) were linked to higher rates of depression. Depressed individuals were also less likely to have been screened for STIs in the past year (aPR: 0.64, 95% CI: 0.43–0.95). No direct association was found between depression and either binge drinking or drug use alone; however, participants reporting both behaviors were nearly 2.5 times more likely to be depressed (aPR 2.49, 95% CI: 1.02–6.05) than those who reported neither or just one of these behaviors.
Ling (2007) (74)	Hong Kong, China	*N* = 89 Street-Based Sex Workers	Cross-sectional study recruited through outreach visits	Suicide and Self-Harm	Clinical Interview according to ICD-10 criteria	Constant environmental threats faced by female sex workers, such as the fear of being infected with HIV and having their identity cards checked frequently, were significantly associated with a higher likelihood of suicidality. Higher educational attainment was associated with a greater likelihood of suicidality. Those with primary school education and an education of high secondary school and above had a significantly higher probability of suicide ideation and attempts than those with no formal education or those who had only completed kindergarten. Those who were cohabitating or divorced were more likely to attempt or consider suicide than those who were single (*p* < 0.05). For instance, those who were cohabitating or divorced had average predicted probabilities of 17.7 and 30.3% for attempting and ever considering suicide, respectively, while those who were single had average predicted probabilities of 0.4 and 9.8% for attempting and ever considering suicide. Respondents who were previously employed had predicted probabilities of 23.2 and 12% for only considering suicide and actually attempting suicide, respectively. On the other hand, those who were previously unemployed or housewives had predicted probabilities of 11.7% and 15.2%, respectively, for ever considering suicide, and predicted probabilities of 0.9% and 2.5%, respectively, for actually attempting suicide.
				Other	The World Health Organization Quality of Life (WHOQOL-BREF), Clinical Interview according to ICD-10 criteria	Fear of being arrested by the police was significantly associated with lower psychological health scores (*p* < 0.05). Those who feared being arrested had average probabilities of 16% for poor psychological health and 7% for excellent psychological health, compared to 1.4% and 34.8%, respectively, among those without such fears. However, the relationship between work environmental threats and mental health was ambiguous in other areas; for example, fear of arrest had a significant but small negative correlation with the incidence of suicide attempts (incidence rate ratio = 0.13, *p* < 0.05). Conversely, fear of HIV infection was correlated with higher psychological health scores (*p* < 0.10). When examining the predicted probabilities of suicide and psychological health, the distributions for average predicted probabilities of poor and excellent psychological health were similar between those afraid of HIV infections and those who were not. Those who were previously employed had higher predicted probabilities (17.6%) of poor psychological wellbeing and lower probabilities of excellent psychological wellbeing compared to those who were previously unemployed or those who were previously housewives. One potential explanation for these findings is that engaging in sex work may be perceived as a greater downward social movement (despite potentially higher financial rewards) for those who were previously employed compared to those who were previously unemployed.
Hong (2007) (28)	Guangxi, China	*N* = 454	Cross-sectional study, recruited from restaurants, barbershops, and hair-washing rooms from three geographic locations: the county seat, a recently established development zone in rural–urban conjunction, and one rural township	Suicide and Self-Harm	Clinical Interview according to ICD-10 criteria	No significant correlation was found with suicidal thoughts across any demographic factors. Similarly, suicide attempts were not significantly associated with most demographic characteristics, except for educational level and cohabitation status. Specifically, women who attempted suicide had, on average, less education (5.6 years compared to 6.7 years, *p* < 0.05) and were more likely to live with their husband or boyfriend (18.40% compared to 6.2%, *p* < 0.01). Those who had attempted suicide began sexual activities at a younger age compared to those who had not. Female sex workers employed in hair salons, including barbershops or hair-washing rooms, exhibited higher instances of both suicidal ideation and attempts than those working in restaurants. Additionally, those reporting suicidal thoughts or attempts often cited relationship breakdowns, deception, or coercion as their reasons for entering sex work. They were also more likely to have one or more steady sexual partners, to have faced sexual coercion in the last six months, or to have had a sexually transmitted disease (STD) history. Conversely, female sex workers without suicidal ideation or attempts tended not to have steady partners and attributed their entry into sex work to financial necessities or peer influence. Four factors were significantly associated with suicidal ideation: being deceived or forced into commercial sex (OR = 3.95, 95% CI = 1.45–10.74), alcohol intoxication (OR = 202, 95% CI = 1.14–3.58), dissatisfaction with life (OR = 2.16, 95% CI = 1.16–4.05), and having stable sexual partners (OR = 2.45, 95% CI = 1.16–5.17 for having one stable partner and OR = 4.34, 95% CI = 1.84–1024 for having multiple stable partners). Four factors were significantly associated with suicide attempt: becoming a female sex worker because of financial needs (OR = 0.24, 95% CI = 0.09–0.58), influence from peers as a reason for becoming a sex worker (OR = 0.10, 95% CI = 0.01–0.81), experience of sexual coercion in the past 6 months (OR = 2.92, 95% CI = 1.31–6.50), and having multiple stable sexual partners (OR = 3.21, 95% CI = 4.89–15.11).
Shahmanesh (2009) (79)	Goa, India	*N* = 326	Cross-sectional study, recruited in cooperation with the largest HIV nongovernmental organization in Goa via respondent driven sampling	Suicide and Self-Harm	The Kessler 10 (K10)	Upon adjusting for socioeconomic factors, belonging to the Kannan ethnicity (originating from Karnataka) and having at least one child significantly decreased the likelihood of a reported suicide attempt. Previous work in the Baina red-light district correlated with a reduced risk of attempting suicide, while having a higher number of regular customers increased the risk. Experiences of physical and verbal abuse from intimate partners or others, along with sexual violence, were linked to suicide attempts. Participation in HIV prevention programs was associated with a decreased likelihood of suicide attempts, but no significant correlation was found with STIs. Intimate partner violence, violence from others, entrapment, and having regular customers were all significant predictors of suicide attempts. Conversely, Kannad ethnicity, recent HIV prevention interventions, and parenthood were linked to a reduced chance of attempting suicide. The inclusion of mental health scores revealed a direct association with suicide attempts, indicating that poorer mental health status significantly increases the risk.
Teixeira (2017) (77)	Porto, Portugal	*N* = 52 Street-Based Sex Workers	Cross-sectional convenience sample, contacted the participants through intermediaries working in five nongovernmental organizations (NGOs)	Suicide and Self-Harm	Suicidal Ideation Questionnaire (SIQ), Social Support Satisfaction Scale	There was a moderate negative correlation between perceived social support and suicidal ideation, indicating that women who felt that they had less social support were more likely to experience suicidal ideation. Drug use was also associated with perceived social support and suicidal ideation. Women using drugs reported that they had less social support and higher levels of suicidal ideation than non-drug users.
Farley (2016) (86)	Minneapolis, Duluth, and Bemidji, Minnesota, USA	*N* = 105 American Indian/Alaska Native (AI/AN) Women in Sex Work from street prostitution (85%); private residences (83%); private parties, hotels, or nightclubs (69%); and bars (68%)	Cross-sectional study, recruited through ads and via snowball or chain referral sampling	Ptsd	The Dissociation subscale of Briere’s Trauma Symptom Checklist (TSC-40), the Post-traumatic Stress Disorder Checklist (PCL)	It was observed that women suffering from PTSD were more prone to classify their health as poor or fair, and significantly less likely to describe it as very good or excellent, in comparison to women not afflicted by PTSD, as demonstrated by a chi-square test result of *χ*^2^ (2, N = 103) = 8,244, *p* = 0.016. In a separate analysis of PTSD’s individual aspects, the recurrence of traumatic memories or flashbacks did not show a significant link with self-assessed health status. Nevertheless, individuals experiencing symptoms of avoidance due to PTSD had a higher likelihood of reporting their health as poor or fair, and a lower likelihood of reporting it as good or excellent, compared to those without PTSD, as indicated by a chi-square test result of *χ*^2^ (2, N = 103) = 6,508, *p* = 0.039. Similarly, women exhibiting symptoms of autonomic nervous system hyperarousal associated with PTSD were more inclined to report their health as poor or fair, and less likely to report it as good or excellent, relative to those without PTSD, evidenced by a chi-square test result of *χ*^2^ (2, N = 103) = 7,362, *p* = 0.025
Alschech (2020) (89)	USA and Canada	*N* = 314 female sex workers advertising their services online (27.40%), selling sex in informal venues such as hotels and clubs (24.80%), or selling sex on the street (8%)	Cross-sectional study, recruited online via E-Mail and from venues	PTSD	The PTSD Checklist (PCL)	Average scores on the PCL differed notably based on the locations where sex was sold. Individuals who marketed their services online independently exhibited significantly reduced scores (35.05) compared to those working in brothels (50.46; *p* < 0.01, Cohen’s d = 1.56). Moreover, workers who identified as White had a significantly lower average score (27, 28) than those identifying as Indigenous (51.74; *p* < 0.01, Cohen’s d = 1.11). While there were no significant differences in mean control over working conditions across the racial groups, workers advertising independently online reported significantly more mean control over their working conditions (73.7%) than workers in brothels, who reported a mean of 61.87% control over their working conditions (Cohen’s d = 0.76). The occurrence of violence from clients was the sole factor not directly linked to posttraumatic stress in a significant manner. The perception of workers regarding their clients’ sense of sexual entitlement emerged as the most potent predictor of traumatic stress, serving as a mediator for the impact of client violence on traumatic stress. This mediator role extended to the effects of identifying as White and marketing sex services online. Discrimination experiences were significant predictors of traumatic stress variations both directly and indirectly, with the degree of control over working conditions playing a crucial role in moderating the relationship between discrimination and traumatic experiences.
Stoebenau (2023) (91)	eThekwini, South Africa	*N* = 644 38.50%, *N* = 248 reported no exchange sex in the past year 17.60%, *N* = 113 reported only transactional sex with a main partner (but no other form of exchange sex) 34.50%, *N* = 222 reported transactional sex with a casual partner (but not sex work) 9.50%, *N* = 61 were female sex workers	Baseline data from intervention study (RCT), recruited through convenience sampling from 34 clusters	PTSD	The Harvard Trauma Questionnaire (HTQ)	Women reporting transactional sex with a causal partner or those reporting sex work were significantly more likely to report PTSD (aOR188, 95% CI: 1.14–3.09 transactional sex with causal partner, aOR: 2.99, 95% CI: 1.52–5.85 sex work), women reporting sex work reported worse health outcomes for PTSD than those engaged in transactional sex with casual partners (but not sex work), although because of small sample sizes the differences were not statistically significant.
Salina (2016) (92)	Chicago, USA	*N* = 200 female sex workers with Substance Use Disorder	Cross-sectional study, recruited participants from multiple substance abuse treatment sites throughout Chicago, the surrounding suburbs and Northern Illinois as well as Cook County Jail	PTSD	The Trauma Symptom Checklist-40 (TSC-40), Personal Progress Scale-Revised (PPSR)	For women who had never engaged in the sex trade, their trauma scores were influenced by whether they had experienced coercion into sexual activities at any point in their lives. Those who had been coerced showed significantly higher trauma scores (mean = 33.17, standard error = 3.41) compared to those who had not faced coercion (mean = 18.68, standard error = 3.02). However, among women who had engaged in the sex trade, the presence or absence of sexual coercion in their lifetime did not lead to significant differences in trauma scores. Furthermore, the analysis revealed no significant variation in trauma symptom levels between women who had experienced sexual coercion, regardless of their involvement in the sex trade. Similarly, there was no significant difference in trauma symptom levels among women who had not experienced sexual coercion, irrespective of their engagement in the sex trade.
Lichtwarck (2023) (100)	Dar es Salaam, Tanzania	*N* = 470	Baseline data from a quasi-experimental intervention study, recruited participants using respondent-driven sampling (RDS)	Alcohol use	The Alcohol Use Disorders Identification Test (AUDIT)	The study found that participants with a history of arrest/incarceration during the past 12 months had a 55% higher prevalence of harmful drinking than those who had not been incarcerated (aPR-adjusted prevalence ratio: 1.55, 95% CI: 1.27 – 1.84). Female sex workers who had experienced gender-based violence during the past 12 months had an aPR of 1.31 (95% CI: 1.06–1.56) compared to those who had not. Moreover, mobility to another city for sex work was independently associated with a 36% increased prevalence of harmful alcohol use as compared to participants who had not traveled (aPR, 1.36. 95% CI: 1.11–1.61)
Boog (2023) (114)	Rotterdam, Netherlands	*N* = 98 *N* = 25 sex workers with drug use disorder (DUD) *N* = 25 sex workers without DUD *N* = 23 women with DUD who were not sex workers *N* = 25 women without DUD who were not sex workers	Cross-sectional study, recruited from inpatient and outpatient facilities, as well as from night clubs, private houses and massage parlors	PTSD	The Traumatic Experiences Checklist (TEC)	The DUD−/SW− group scored significantly lower than the other three groups on the TEC total variable, indicating a lower general level of experienced trauma. The DUD−/SW+ group obtained a significantly lower score on TEC total than the DUD+/SW+ group.
Griffith (2013) (102)	Los Angeles, California, USA	*N* = 176 Performers in the Adult Entertainment Industry, 58 performers (32.9%) identified as heterosexual and 118 (67.1%) identified as bisexual	Cross-sectional, convenience sampling conducted at the Adult Industry Medical Healthcare Foundation (AIM)	Substance Use and Dependence	The Short Michigan Alcohol Screening Test (SMAST)	Across the 15 categories of drugs, there were three differences, all showing a higher percentage of bisexual actresses having tried a particular drug compared to heterosexual actresses. The differences were in alcohol use [*χ*^2^(1) = 6.71, *p* < 0.05; bisexual *n* = 116, 98.3%; heterosexual *n* = 52, 89.7%], marijuana use [*χ*^2^(1) = 5.22, *p* < 0.05; bisexual *n* = 99, 83.9%; heterosexual *n* = 40, 69%], and hallucinogen use [*χ^2^*(1) = 4.9, *p* < 0.05; bisexual *n* = 53, 41.4%; heterosexual *n* = 16, 24.6%]. The second step examined drug use across the 15 categories during the last 6 months, revealing one significant finding for cocaine use [t (29) = −234, *p* < 0.05], with heterosexual porn actresses using cocaine more often (M = 1, SD = 0.93) than bisexual actresses (M = 0.53, SD = 0.74). Based on these data, there are relatively few differences in alcohol and drug use between bisexual and heterosexual porn actresses. The differences are mixed: more bisexual actresses had tried alcohol, marijuana, and hallucinogens, but heterosexual actresses had higher rates of recent cocaine use.
Gilchrist (2005) (42)	Glasgow, Scotland	*N* = 176 sex workers, *N* = 89 non sex workers	Cross-sectional study with control group, recruited via convenience sampling from a drop-in center, a crisis center and a methadone medical specialist service	Other	The Revised Clinical Interview Schedule (CIS-R)	Emotional abuse as an adult (OR: 8, 95% CI: 2.4–27.2), 12-month dependence on cannabis (OR: 7.9, 95% CI: 1.9–32.9) and self-reported lifetime eating disorder (OR: 7.1, 95% CI: 1.3–40.1) were significantly associated with a score of 18 for non-sex workers.
Rössler (2010) (16)	Zurich, Switzerland	*N* = 193 *N* = 66 from street sex work *N* = 53 from brothels and cabaret clubs *N* = 42 from studios who reported sex work to be not burdening or slightly burdening *N* = 30 from studios who described their work as highly burdening	Cross-sectional study, utilized quote sampling	Other	The WHO Composite International Diagnostic Interview (M-CIDI2.1)	Logistic regression analyses identified the following correlates of 1-year prevalence rates of mental disorders: cultural background, work setting, subjectively perceived and objectively experienced burden associated with sex work and subjectively perceived social support. Participants from countries other than Switzerland, Western, and Eastern Europe showed a higher likelihood of outcomes (OR: 3.84, 95% CI: 1.61–9.20, *p* = 0.003). Having no one to trust was associated with an increased likelihood of the outcomes (OR: 2.13, 95% CI: 1.10–4.15, *p* = 0.025), whereas being supported by family was associated with a lower likelihood (OR: 0.23, 95% CI: 0.10–0.49, *p* < 0.0001). Feeling excluded by acquaintances or society was significantly associated with the outcomes. Working days per week and customers per week were also associated with an increased likelihood of the outcomes (OR: 1.25, 95% CI: 1.04–1.50, *p* = 0.016 for working days; OR: 104, 95% CI: 1.01–1.07, *p* = 0.022 for customers). Auxiliary income was associated with a lower likelihood of the outcomes (OR: 0.37, 95% CI: 0.2–0.71, *p* = 0.003). Experiences of violence within and outside the red-light milieu showed significant associations with the outcomes. Violence in the red-light milieu (OR: 3.05, 95% CI: 1.53–607, *p* = 0.002), rape in the red-light milieu (OR: 2.22, 95% CI: 1.06–4.65, *p* = 0.035), and rape out of the red-light milieu (OR: 2.07, 95% CI: 1.11–3.89, *p* = 0.023) were all significantly correlated with the reported outcomes. The sum score for burden by sex work showed a significant association with the outcomes (Mean: 6.41, 95% CI: 3.44–11.94, *p* < 0.0001).
Buttram (2014) (119)	Miami, Florida, USA	*N* = 562 Street-Based Sex Workers, who were African American and used cocaine, crack, or heroin three or more times a week in the past 30 days	Baseline data from a randomized clinical trial, recruited through targeted sampling and outreach strategies	Other	The Personal Mastery Scale, the Global Appraisal of Individual Needs (GAIN) the TCU Drug Screen II, the General Victimization Scale (GVS)	A history of foster care, homelessness in the past 90 days, substance dependence, severe mental distress, risk of HIV, and severe victimization were all significantly associated with a reduced likelihood of exhibiting high personal mastery. Conversely, having an education of 12 years or more, access to transportation, and increased social support were linked to a higher probability of high personal mastery. Severe mental distress and the risk of HIV transmission continued to be significantly associated with lower chances of high personal mastery. Conversely, factors that positively influenced high personal mastery included having an education of 12 years or more and receiving greater social support.
Brody (2016) (121)	Cambo.dia	*N* = 657 44.40% worked at Karaoke Bars, 30.60% at restaurants, 25% worked in other places, e.g., beer gardens, nightclubs, bars, massage parlors and streets	Cross-sectional study, recruited from entertainment venues and hotspots	Other	The brief screening version of the Childhood Trauma Questionnaire (CTQ), short version of the General Health Questionnaire (GHQ-12)	Women with higher levels of psychological distress were significantly more likely to have consumed a full glass or can of alcohol (90.9% vs. 95.1%, *p* = 0.04), been forced to drink at work (19.8% vs. 38.3%, *p* < 0.001), and used illicit drugs (0.5% vs. 3.2%, *p* = 0.01) in the past 3 months. After controlling for confounding factors, these women were significantly more likely to rate their overall health as poor (AOR = 1.88, 95% CI 1.20 to 2.94), rate their quality of life as poor (AOR = 2.39, 95% CI 1.47 to 3.87), have had suicidal ideation in the past 3 months (AOR = 2.41, 95% CI 1.45 to 3.76), and rate their HIV risk as higher than the general population (AOR = 0.48, 95% CI 0.31 to 0.74). They were also more likely to have been forced to drink at work in the past3 months (AOR = 1.77, 95% CI 1.19 to 2.62), have had clients requesting not to use a condom (AOR = 3.48, 95% CI 1,14 to 10,62), been unable to find condoms when needed (AOR = 0,64, 95% CI 0,45 to 0,93), had a family member who said hurtful things to them during childhood (AOR = 1,84, 95% CI 1,24 to 2,75), and had a parent or guardian who had been physically abusive (AOR = 193, 95% CI 1.34 to 2.82).
Tchankoni (2020) (94)	Togo, Africa	*N* = 952	Cross-sectional bio-behavioral study, recruited in drug-dealing/consumption locations and brothels (licensed or not)	Other	The Kessler PD Scale (K10)	Respondents aged 25 and older were more likely to have severe/moderate or mild psychological distress (PD) than younger respondents (*p* = 0.026). A positive HIV serological status was identified as a risk factor for PD. The likelihood of PD was higher among hazardous drinkers (aOR = 152; 95% CI: 1.22–1.87) compared to non-drinkers. Individuals with a secondary school education or higher (college/university level) were less likely to have severe/moderate or mild PD compared to those who only completed primary school (*p* < 0.001). Compared to drug users (DU), female sex workers (aOR = 0.55; 95% CI: 0.43–0.68) and men who have sex with men (aOR = 0.33; 95% CI: 0.24–0.44) were less likely to have severe/moderate or mild PD.

### 3.2 Study selection

**Figure 1:**
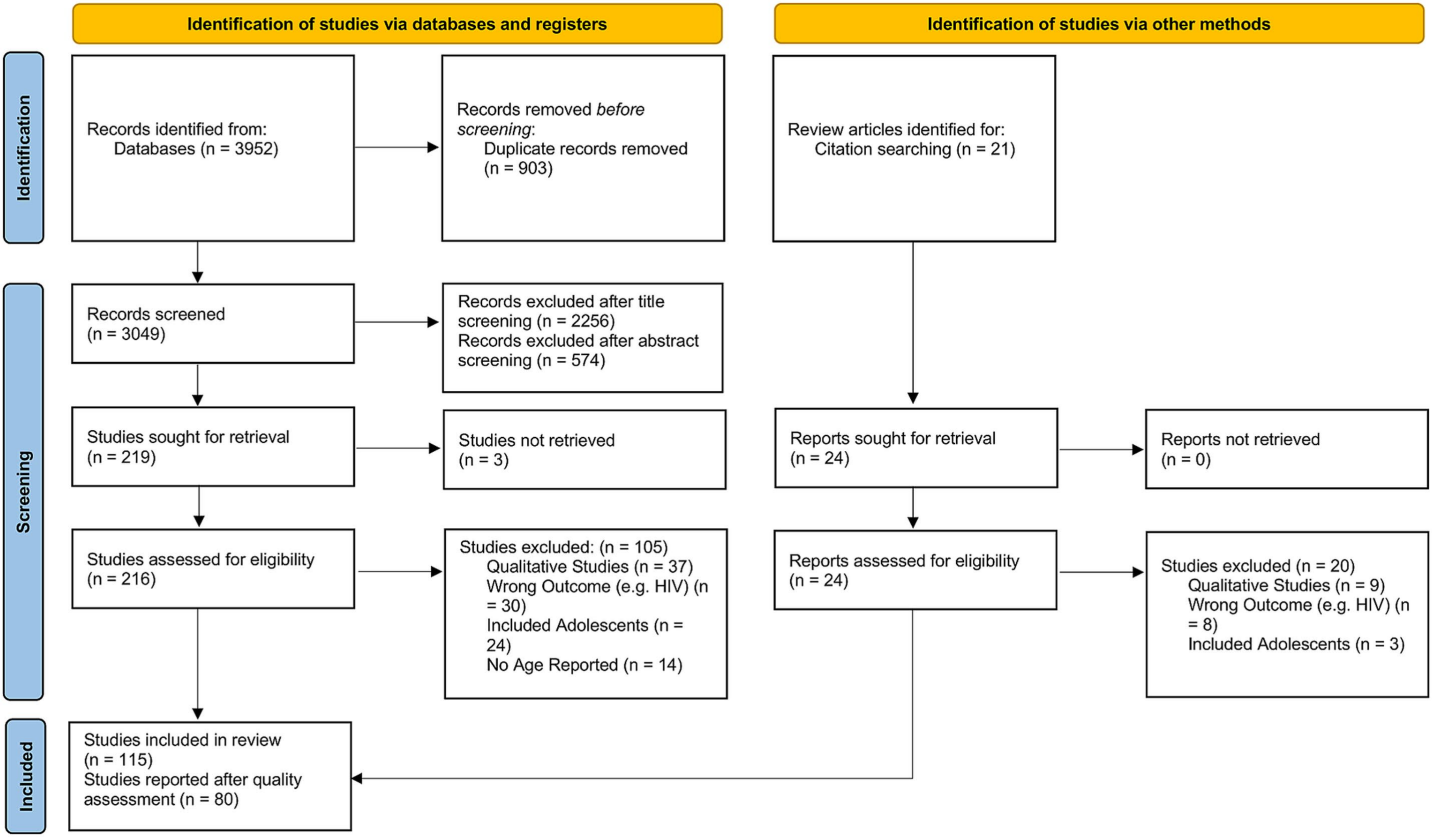
PRISMA flowchart summarizing the study selection process.

A total of 3,952 studies were identified. First, duplicates (*n* = 903) were removed, leaving 3,049 studies for abstract and title screenings. Of these, 2037 studies were excluded because they did not meet the inclusion criteria. From the remaining literature, 219 studies were eligible for full-text analysis. Three studies could not be retrieved (30–32). A total of 105 studies were excluded from the analysis for different reasons (i.e., focused on STDs or did not report the age, qualitative instead of quantitative measurements, etc.) the remaining 111 studies were included, and for 21 review articles citation tracking was used resulting in 24 studies for further full-text screening. A total of 20 studies were excluded from the analysis resulting in a total of 115 studies that were finally included. Figure 1 summarizes the selection process.

### 3.3 Quality assessment

After quality assessment, a total of 80 remaining studies were analyzed for the systematic review. Several studies (*n* = 23) were excluded from the systematic review due to the absence of standardized instruments for data collection. One publication was excluded because the used instruments were not described. Additionally, some publications were not included because they presented secondary data analyses with the original data already reported in previous publications (*n* = 7). We included only the original publications ensuring prevalences and risk factors were not reported twice. Four studies scored below 3 points on the Newcastle Ottawa Scale and therefore were excluded from the report according to our review methodology. Supplementary Table 2 presents an overview of the studies that did not meet the quality standards. The scores of the 80 included studies ranged from 3 to 9, with most studies scoring between 4 and 6. The distribution indicates that the majority of studies met a moderate level of quality, with a few outliers achieving higher scores of 7 or 9, while some scored lower, suggesting a more variable overall quality. Supplementary Table 3 provides an overview of the included studies and the quality scores. The included studies comprise a total of 24,675 individuals.

### 3.4 Geographic distribution of included studies

**Figure 2:**
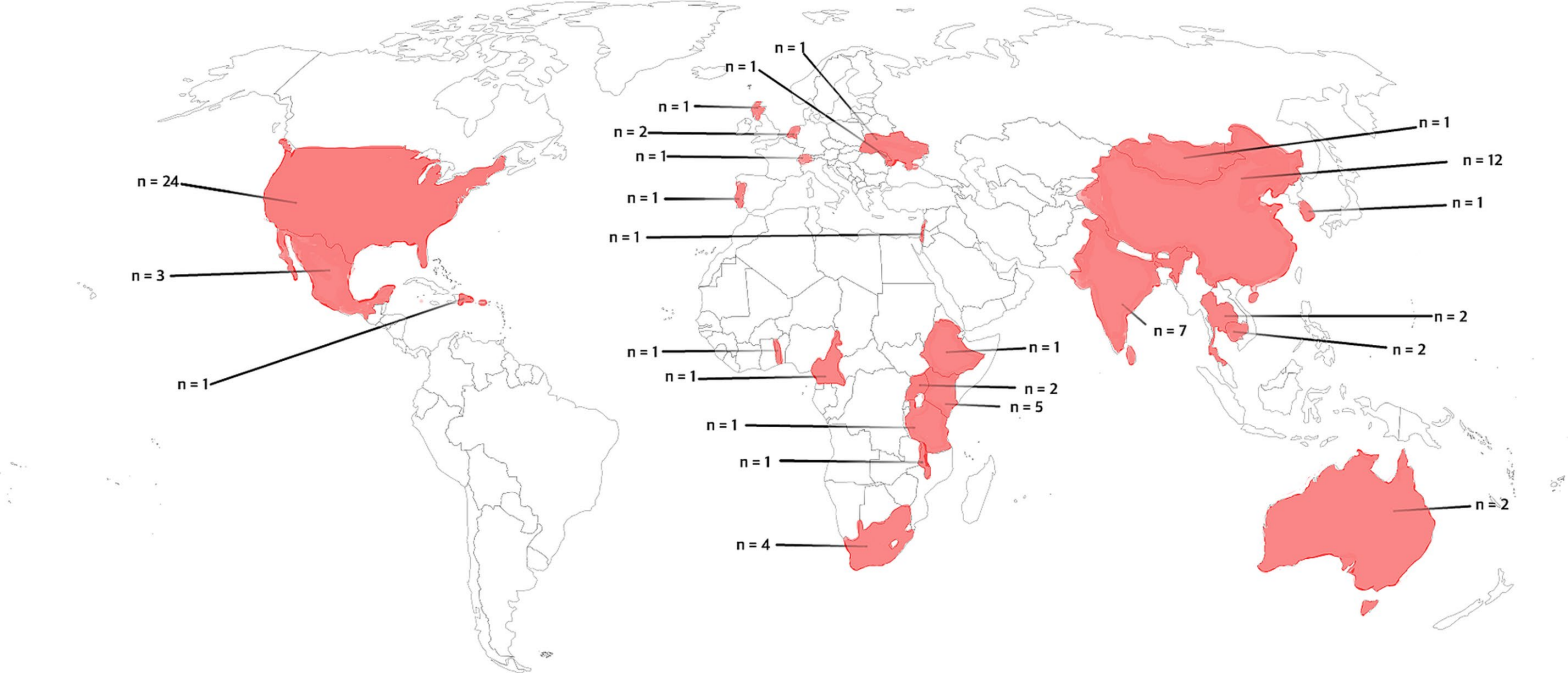
Global distribution of included studies.

Figure 2 shows the global distribution of identified studies. The studies originate from 26 different countries. The majority of studies stemmed from the United States (*n* = 24), followed by China (*n* = 12), India (*n* = 7) and Kenya (*n* = 5). Four studies were conducted in South Africa and three in Mexico. Two studies originated from Australia, Cambodia, Thailand, the Netherlands, and Uganda. Single studies were identified from Scotland, Switzerland, Israel, Portugal, Mongolia, Malawi, Cameroon, Ukraine, Togo, Lebanon, the Dominican Republic, Tanzania, Puerto Rico, Ethiopia, and Moldova.

### 3.5 Subclinical anxiety and anxiety disorders

#### 3.5.1 Prevalence of subclinical anxiety and anxiety disorders

The prevalence of subclinical anxiety and anxiety disorders among sex workers has been studied across various global locations and populations through 14 studies (see Table 1), with four studies stemming from the USA and 10 single studies from Lebanon, Kenya, the Dominican Republic, China, Ethiopia, Scottland, Switzerland, India, Thailand and Moldova. A large proportion of the research utilized cross-sectional surveys to collect data, with several studies forming part of baseline data collection for broader intervention studies. For instance, Edwards et al. (26) and Risser et al. (33) both analyzed baseline data from intervention studies focusing on substance abusers who engage in sex work. Slim et al.’s work (34) differed as a case–control study, recruiting participants from a prison setting, while Beksinska et al. (35) utilized mixed methods. The instruments used to measure anxiety varied across studies. They include, e.g., the anxiety scale of the Drug Abuse Treatment AIDS Risk (DATAR) questionnaire (36), the Hamilton Anxiety Rating Scale (HAM-A) (37), the Generalized Anxiety Disorder tool (GAD-7) (38), and modules from the Hospital Anxiety and Depression Scale (HADS) (39).

A review of the literature reveals a wide range of anxiety prevalence among sex workers, from the lowest reported rate of 5.2% by Rossler et al. (16) for generalized anxiety disorder in Zurich, Switzerland to the highest of 75.8%, among migrant sex workers in Mae Sot, Thailand reported by Decker et al. (40).

In the lower prevalence range, Rossler et al. (16) reported rates for panic disorder (8.8%), simple phobia (17.6%), and agoraphobia without panic (2.1%), illustrating a spectrum of anxiety disorders among sex workers. Similarly, Iaisuklang et al. (41) found a prevalence of 8% for generalized anxiety disorder. Middle-range prevalences include findings from Gilchrist et al. (42) in Scotland, with 26% of sex workers experiencing phobias and 24% panic disorders. At the higher end of the spectrum, Ghafoori et al. (43) reported a 56.9% prevalence among survivors of sex trafficking, which was still lower than among the two comparison groups, namely survivors of domestic violence (70%) and sexual assault (79.3%).

#### 3.5.2 Risk and resilience factors for anxiety

Across the 7 identified studies, a range of risk and resilience factors for anxiety have been identified (Table 2). Beksinska et al. (35) found that in Kenya, a higher prevalence of Adverse Childhood Experiences (ACEs) was associated with anxiety, with factors such as experiencing violence and war in childhood, missing meals due to lack of food, and non-consensual sexual debut being significant predictors. However, social support seemed to play a protective role. Mac Lin et al. (23) reported that sex work related police harassment significantly increased the likelihood of scoring abnormal on the anxiety-specific module of the HADS-A. Zhai et al. (27) identified that married female sex workers, those living in the county with low education, and those with social support were less likely to report anxiety. Quarantining due to the pandemic was an independent risk factor (27). Kelton et al. (44) found that greater current financial concern and lower social capital were linked to higher anxiety symptoms. Yesuf et al. (45) observed that street sex workers reported higher anxiety levels compared to those working from home, with khat use, violence, stigma, and tobacco use being significant predictors of anxiety. Lastly, Decker et al. (40) noted that anxiety was more prevalent among migrant sex workers who experienced fraud, force, or coercion.

### 3.6 Depression

#### 3.6.1 Prevalence of depression

In this section, we analyze the prevalence of depressive symptoms among sex workers through 41 studies conducted by various authors across different locations globally (see Table 1). Among these, most of the studies employed a cross-sectional design, utilizing both recruitment through agencies and snowball sampling strategies. The instruments and measures used to assess symptoms of depression varied across studies, including, e.g., the Center for Epidemiologic Survey-Depression Scale (CES-D) (46), the Beck Depression Inventory-II (BDI-II) (47), and the depression subscale from the Brief Symptom Inventory (BSI) (48).

This review presents a broad spectrum of prevalences of depressive symptoms among female sex workers, highlighting the complexity of mental health within this demographic. The analysis spans from the lowest reported prevalence of 3.3% for major depression (49) to the highest of 100% in a subgroup of female sex workers living with HIV reported by Mac Lin et al. (23).

Sherwood et al. (50) and Iaisuklang et al. (41) reported relatively low prevalences of 10.3 and 9% respectively, suggesting areas or conditions under which sex workers may experience comparatively better mental health outcomes. Contrasting these findings, Bhardwaj et al. (51) found 33.2%, indicating a relatively moderate level of depression among sex workers on the spectrum of included studies. Mid-range prevalences are represented by studies such as in the works of Rossouw et al. (52), Grudzen et al. (53) Abelson et al. (25) and Hong et al. (54). At the higher end of the spectrum, findings from Gilchrist et al. (42), and Coetzee et al. (55) reported prevalences of 70% and 68.7%.

Some studies focused on specific subgroups or included a comparison group. Ulibarri et al. (56), e.g., found that 86% of a subgroup of female sex workers who reported injection drug use exceeded the cutoff score for depression, highlighting the intersectionality of drug use and mental health challenges in this population (57). Other studies, such as those by Slim et al. (34) in Lebanon, and Risser et al. (33) in Houston, Texas, USA, showed higher depression scores among sex workers compared to non-sex workers, further evidenced by the findings of Kim et al. (58) in Chicago, Illinois, where sex workers showed a higher prevalence (81%) compared to persons engaged in care and service work.

#### 3.6.2 Risk and resilience factors for depression

This review identified a wide range of risk and resilience factors for depression among sex workers, underscoring the complexity and diversity of experiences within this population (see Table 2). The studies span multiple continents and encompass a variety of study designs (59, 60) as well as socioeconomic, behavioral, and interpersonal dimensions.

Socioeconomic factors, stigma, and experiences of violence emerged as the most extensively studied predictors of depression. Krumrei-Mancuso et al. (5) highlighted motivation for entering sex work as a significant determinant of depression, with economic motivation linked to higher depression levels. This contrasts with those entering for pleasure or a combination of reasons, suggesting the critical role of agency and motivation in mental health. Moreover, confidence in finding alternative employment served as a protective buffer against depression. Social support emerged as a strong protective factor in several studies, including Carlson et al.’s (61) work. Conversely, stigma and sexual violence were consistently identified as significant risk factors (24, 25, 35, 62, 63). A good relationship with gatekeepers, individuals who manage the establishments and/or sex workers, was a protective factor for depression (64).

Besides individual socioeconomic factors, psychiatric co-morbidities and systemic factors were identified as factors influencing the depression rates of sex workers. Early initiation into sex work, as suggested by MacLean et al.’s (65) study, and substance use severity (66) were implicated as contributors to higher depression rates. Coetzee et al. (67) and Stockton et al. (63) found that pregnancy, child loss, and food insecurity further complicate the relationship between personal circumstances and depression among female sex workers. Racial disparities and the impact of incarceration on mental health were noted in the United States by Murnan et al. (68) and Kim et al. (58), respectively, while living with HIV, and verbal abuse were associated with major depression in Northern Uganda (69). (Intimate partner) violence, alcohol use, Khat use, and age were identified as significant risk factors of depression (29, 45, 70). Institutional factors such as police harassment (23) and environmental factors like mobility and working conditions (71) were also recognized as influencing depression levels.

### 3.7 Suicide and self-harm

#### 3.7.1 Prevalence of suicide and self-harm

The research designs applied in the 9 identified studies varied, including cross-sectional studies with and without control groups (see Table 1). Recruitment strategies ranged from reaching out to participants through agencies, community outreach, and specific locations such as streets and restaurants, to collaborations with health programs. Several instruments were utilized across the studies to measure suicide and self-harm behaviors as well as mental health indicators. These included the Revised Clinical Interview Schedule (CIS-R) (72) and the Kessler 10 (K10) (73), among others.

The prevalence of suicidal behaviors among sex workers varies widely from lower incidences reported in Soweto, South Africa (67), with 3.6%, to notably higher rates in other regions and among specific subgroups. Multiple studies were focused on street-based and/or drug-using sex workers, who seem to display higher rates of suicidality. Gilchrist et al. (42) reported that 39% of drug-using sex workers engaged in deliberate self-harm, and 53% had attempted suicide, highlighting the severe mental health concerns within this demographic. Roxburgh et al. (9) found even more alarming figures in Sydney, Australia, where 74% of street-based female sex workers had contemplated suicide, and 42% had attempted it at least once, underscoring the critical mental health risks faced by sex workers. Ling et al. (74) identified that 25.8% of street-based sex workers had suicidal thoughts, and 6.7% reported attempting suicide. Similarly, Fang et al. (75) found that 14% considered suicide in the past 6 months, with 8% attempting suicide within the same timeframe. Zhang et al. (76) reported a prevalence of suicidal behavior at 9.5%. Teixeira et al. (77) reported that 44.2% of street-based participants stated having made at least one suicide attempt (78).

#### 3.7.2 Risk and resilience factors for suicidality

The collection of studies highlighted here examines the risk factors for suicidality among sex workers in various geographical locations (see Table 2). Socioeconomic factors, co-morbidities, violence and institutional factors such as law enforcement were identified as risk factors for suicidality. Ling et al. (74), e.g., identified frequent identity card checks as significant contributors to increased suicidality risk among sex workers. Hong et al. (28) reported that key factors linked to suicidality included educational level, cohabitation status, relationship issues, sexual coercion, and history of sexually transmitted diseases (STDs), highlighting the impact of interpersonal and health-related stressors. Shahmanesh et al. (79) in Goa observed that age, ethnicity, number of children, and length of residence were influential, with violence, entrapment, and having regular customers also noted as predictors of suicide attempts. Protective factors included non-migrant ethnicity, participation in HIV prevention interventions, and parenthood, indicating the potential of community and familial support systems in mitigating suicide risk. Like in the case of previously reported mental conditions, stigma emerged as risk factor for suicidality. Zhang et al. (80), e.g., discovered that violence from stable partners and clients, psychological abuse, and higher levels of stigma significantly increased the likelihood of suicidal ideation or attempts.

### 3.8 PTSD

#### 3.8.1 Prevalence of PTSD

Our systematic review delved into the prevalence of Post-Traumatic Stress Disorder (PTSD) among sex workers, incorporating 16 studies from diverse geographic locations (see Table 1). Among the included studies, a majority were cross-sectional in design, focusing on assessing the current prevalence and conditions among sex workers. The results of these studies highlighted a significant variation in PTSD prevalence among the sex worker populations studied, with the instruments used to measure PTSD symptoms including, e.g., the PTSD Checklist (PCL-5) (81), the Composite International Diagnostic Interview (CIDI) (82), and the Davidson Trauma Scale (DTS) (83).

PTSD prevalences were notably linked to specific subgroups, namely street-based, drug-using sex workers and survivors of human trafficking. Roxburgh et al. (9) highlighted that nearly half (47%) of street-based female sex workers met DSM-IV criteria for lifetime PTSD prevalence, with 31% meeting the criteria for current PTSD. Cigrang et al. (84) observed a 66% PTSD prevalence among sex workers arrested for at least one drug offense. Hendrickson et al. (71) reported a 53% PTSD prevalence among street-based sex workers, while Suresh et al. (85) found that 42% had moderate PTSD symptoms and 12% had extreme symptoms. Farley et al. (86) focused on street sex workers, finding that 52% met all criteria for a PTSD diagnosis. Ostrovschi et al. (49) examined women returning to Moldova after being trafficked, noting a decrease in PTSD diagnoses from 48.3% in the crisis intervention phase to 35.8% in the re-integration phase.

Some studies implemented comparison groups. Ghafoori et al. (43) compared sex trafficking survivors with domestic violence and sexual assault victims, finding lower PTSD rates among the first (61.2%) compared to 70.5% and 83.6% in the second and third group, respectively. Edwards et al. (26) found that among African American crack abusers, those who traded sex were more likely to report PTSD symptoms than non-sex workers. Jung et al. (87) compared former sex workers to activists and a control group, finding the highest prevalence of PTSD symptoms among former sex workers.

The studies varied widely in sample sizes. Broader studies like Beksinska et al. (35) (*n* = 1,003) reported a PTSD prevalence of 14.2% among female sex workers, while smaller studies such as Chudakov et al. (88) in Israel (*n* = 55), found a 17% prevalence.

#### 3.8.2 Risk and resilience factors for PTSD

PTSD prevalences were mostly influenced by experience of violence, but socioeconomic factors also played significant roles. Roxburgh et al. (9) found a strong association between adult sexual assault, a higher number of traumas, and current PTSD among female sex workers, with no significant differences noted in child sexual abuse or physical assault at work based on PTSD status (see Table 2). Jung et al. (87) observed that the work duration and age positively correlated with PTSD symptom severity. Suresh et al. (85) reported that violence encountered by sex workers was a key factor in the heightened levels of depression and PTSD, indicating that violence, rather than the sex work itself, contributed significantly to their mental distress. Farley et al. (86) identified a direct correlation between PTSD symptom severity and lower health ratings among American Indian/Alaska Native women in sex work. The fact that PTSD prevalence is associated with the workplace and the specific subgroup, such as drug-using sex workers, is further evidenced by Alschech et al. (89), who observed that online independent sex workers in the USA and Canada had significantly lower PTSD scores compared to brothel workers, and Tomko et al. (90), who noted higher recent benzodiazepine use among women with high PTSD scores. Social circumstances were predictors of PTSD prevalences, as in the case of Stoebenau et al. (91), who found that PTSD likelihood was higher among women reporting sex work or transactional sex with a casual partner compared to those with a main partner only. Contrary to the previously named studies, Salina et al. (92) reported that past sexual coercion did not significantly affect trauma scores among women engaged in the sex trade, presenting a nuanced view of trauma’s role in sex work.

### 3.9 Substance use and substance dependence

#### 3.9.1 Prevalence of hazardous alcohol use and alcohol dependence

In the systematic review section, we delve into the findings of 14 studies that shed light on this topic across a global spectrum (see Table 1). The array of studies investigating alcohol use and dependence among sex workers employs a rich tapestry of study designs and instruments. Predominantly, the research leans toward cross-sectional designs, with notable variations in recruitment strategies that cater to the specific contexts and populations under study. For instance, Pandiyan et al. (66), and Couture et al. (93), employed cross-sectional approaches with participants recruited from either psychiatric hospitals or entertainment venues, respectively, underscoring the diversity in setting and approach. Tchankoni et al. (94) bio-behavioral study, and Rash et al. (95) baseline data from an intervention study illustrate the methodological breadth, ranging from respondent-driven sampling (67) to targeted sampling at various venues (51). A common thread is the utilization of the Alcohol Use Disorder Identification Test (AUDIT) (96) and its concise counterpart, AUDIT-C (97). Other instruments included the Short Michigan Alcohol Screening Test (SMAST) (98), or, e.g., the WHO ASSIST tool (99).

Prevalences ranged from 100 to 0% for alcohol use and dependence. Pandiyan et al. (66) in Bangalore, India, found alcohol use to be universally prevalent among the study’s participants. Couture et al. (93) in Cambodia reported that a significant majority of female sex workers and male clients engaged in hazardous drinking, with an 85% prevalence rate according to the AUDIT-C scale. In Togo, Africa, Tchankoni et al. (94) found that 77% of female sex workers used alcohol at harmful or hazardous levels, with Lichtwarck et al. (100) reporting a slightly lower but still high prevalence rate of 69.6%. Some studies found lower rates of 26.3% and 29.8%, respectively, (51, 101). Stoebenau et al. (91) observed a 45.9% prevalence among sex workers compared to 14% among non-sex workers, highlighting a significant disparity. Griffith et al. (102) found problematic drinking levels among porn actresses to be similar to those of the general population while Rossler et al. (16) reported a 0% prevalence of alcohol dependence.

#### 3.9.2 Prevalence of substance use and dependence (other than alcohol)

The systematic review on substance use among sex workers encompasses findings from 16 studies conducted in diverse locations (see Table 1). The applied study designs include intervention studies utilizing baseline data (103), cross-sectional studies with (42) and without control groups (41), and mixed methods approaches (104). Some studies employed specific recruitment strategies such as venue-based sampling (33) others used health records (95). The instruments used to measure substance use varied across studies, incorporating tools like hair assays for detecting cocaine and opiate use (103), or, e.g., the Diagnostic Interview Schedule for assessing drug dependency and psychiatric diagnoses (105). Instruments such as, e.g., the Addiction Severity Index (ASI) (106), and the Drug Abuse Screening Test–10 (DAST-10) (107) were utilized to quantify the extent of substance dependence and to evaluate the associated risks and health concerns in diverse geographic and sociocultural contexts.

The identified studies were mostly focused on street-based sex workers. Nuttbrock et al. (103) in New York City, USA, found that among street-based sex workers, 92.7% had used cocaine and 44.4% had used opiates in the month before testing. Gilchrist et al. (42) observed a high level of polydrug use, with 79% of sex workers reporting such use in the last 30 days, including 18% for cocaine use and 72% for injecting drugs, with 55% experiencing accidental drug overdoses. Kurtz et al. (104) reported widespread substance use among sex workers, including crack cocaine (73.4%), and marijuana (59.9%), among others. Risser et al. (33) highlighted longer durations and higher frequencies of crack use among current sex workers. Broader studies, such as in the case of Beksinska et al. (101), reported harmful levels of amphetamine (21.5%) and cannabis use (16.8%) among female sex workers, with a minor fraction reporting injecting drug use (0.5%). Some studies reported a comparison group: Vaddiparti et al. (108) observed a disparity in cocaine dependence between sex workers and non-sex workers, while Rash et al. (95) found no significant difference in opioid use disorder between the two groups. While cocaine, opiates, amphetamines, and cannabis were the substances most commonly used by sex workers, only a small fraction appeared to consume psychoactive substances overall. Iaisuklang et al. (41) in Shillong, Meghalaya, India, e.g., reported a small percentage (3%) of female sex workers were diagnosed with non-alcohol psychoactive substance use disorder.

#### 3.9.3 Risk and resilience factors for substance use and substance dependence (including alcohol)

Due to limited literature in this field (6 identified studies), risk factors will not be reported separately for substance use, substance dependence as well as hazardous alcohol use and alcohol dependence (see Table 2). Socioeconomic factors and experiences of violence were identified as the predominant risk factors, though more specific factors, such as genetic predisposition, were also noted. Zhang et al. (109) identified younger age, unmarried status, higher education levels, and employment in alcohol-serving venues as factors increasing the propensity for alcohol-related problems among female sex workers. This group was also at a heightened risk of sexual exploitation and violence by clients, with a correlation noted between the severity of drinking behavior and these risks. Chen et al. (110) found that female sex workers with at least a high-school education, married status, and higher monthly income exhibited higher alcohol risk levels. L’Engle et al. (111) demonstrated the effectiveness of targeted behavioral interventions in reducing drinking levels among moderate-risk drinking female sex workers through a randomized controlled trial. In Cambodia, Couture et al. (93) observed higher rates of unhealthy alcohol consumption among female sex workers employed in entertainment venues and those with children, noting the significant influence of education level on alcohol consumption patterns among male clients. Zhang et al. (112) pointed out that female sex workers with first and second-degree relatives who drank alcohol had higher AUDIT scores, suggesting genetic and environmental contributions to alcohol use disorders. Further research by Beksinska et al. (101) indicated that female sex workers with harmful alcohol use reported higher ACE scores, street living, and experiences of childhood sexual/physical violence, emphasizing the need for early interventions to address these complex interrelations. Similarly, Roxburgh et al. (113) noted that sexual violence was a risk factor for substance abuse. Lichtwarck et al. (100) found that a history of arrest/incarceration, experiencing gender-based violence, and mobility for sex work significantly raised the prevalence of harmful alcohol use among female sex workers. Boog et al. (114) found no statistical differences in drug use intensity compared to non-sex workers when adjusted for age and level of education, suggesting that drug use patterns among sex workers may not be distinctly different from other groups.

### 3.10 Other conditions

#### 3.10.1 Prevalence of personality disorders, eating disorders, schizophrenia, obsessive-compulsive disorder, and other disorders

A variety of instruments have been employed across these studies to measure and evaluate mental health outcomes (see Table 1). These include the composite International Diagnostic Interview (115), the Psychopathy Checklist: Screening Version (PCL:SV) (116, 117), the Mini International Neuropsychiatric Interview (118), the Kessler Scale (73), and clinical interviews based on ICD-10 and DSM 5 criteria. The studies’ designs ranged from cross-sectional with different recruitment strategies, including outreach and agency recruitment, baseline data from intervention studies (119–121), longitudinal studies following up survivors of human trafficking, to cross-sectional studies with control groups (122).

Rossler et al. (16) found a 0% one-year and lifetime prevalence of schizophrenia among sex workers but noted a 10.4% one-year prevalence and an 11.4% lifetime prevalence for somatoform disorders. Obsessive-compulsive disorder (OCD) prevalence rates were both reported at 2.1% for 1 year and lifetime. The one-year prevalence for eating disorders was 5.2%. Jiwatram-Negron et al. (123) highlighted significant mental health issues among female sex workers, with 30% reporting hospitalization for mental health concerns at some point, and sex traders experiencing notably higher rates of hospitalization compared to non-trading women. Edwards et al. (26) identified that female sex workers had a significantly higher mean score on the PCL:SV Psychopathy measure compared to controls. Iaisuklang et al. (41) reported that 9% of participants had been diagnosed with Antisocial Personality Disorder (APD). Ostrovschi et al. (49) found initial diagnoses of Somatization Disorder (2.5%), Adjustment Disorder (13.3%), and Acute Stress Reaction (10%) among survivors of human trafficking during the crisis intervention phase, which were not present during the re-integration phase. Gilchrist et al. (42) reported a compulsion prevalence of 37%, an obsession prevalence of 53%, and a lifetime prevalence of 37% for eating disorders among sex workers.

#### 3.10.2 Meta-analysis of prevalences of mental disease

A random-effects meta-analysis was conducted for “Subclinical Anxiety or Anxiety Disorder” (k = 10), “Depression” (k = 29) and “PTSD” (k = 13). The available literature for other disease prevalences or risk factors was not sufficient to conduct a meta-analysis. All studies included in the meta-analysis captured the prevalence of current symptoms, not the lifetime prevalence. While the studies on depression and PTSD captured symptoms that were indicative of the diagnosis, the studies on anxiety focused either on screening for anxiety disorders according to ICD or DSM (e.g., through the Composite International Diagnostic Interview) or on a screening for elevated anxiety levels, that indicate the need of further clinical diagnostics and treatment (e.g., through the Beck Anxiety Inventory). A subgroup analysis was conducted for each condition testing the legal status of sex work (using the categories “legal,” “partially legal” and “illegal”), the sub-population, and economic conditions (GDP per Capita) as mediators. Additionally, we conducted a subgroup analysis for each condition to examine whether the method of assessment (clinical interview versus other assessment tool) influenced the reported prevalence rates. Publication bias was assessed through funnel plots and the Egger’s test.

### 3.11 Anxiety

#### 3.11.1 Overall findings on anxiety prevalence

**Figure 3:**
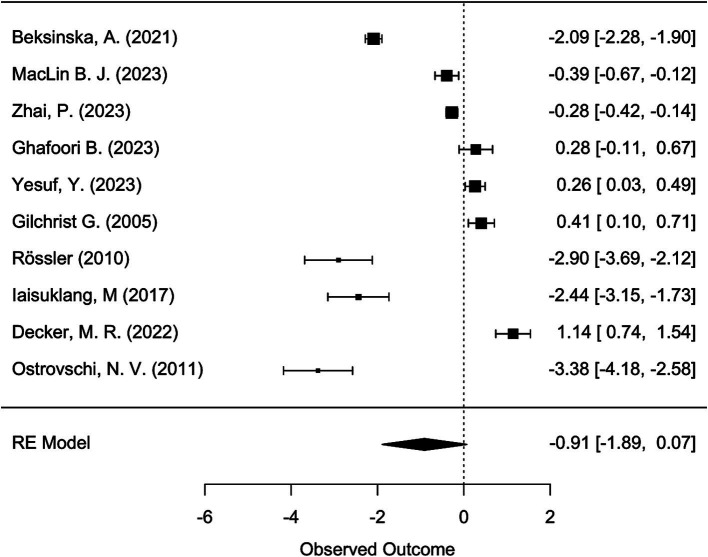
Forest plot showing the logit-transformed prevalence of subclinical anxiety and anxiety disorder and calculated confidence intervals across the included studies.

The random-effects model analysis underscored a profound heterogeneity among the studies (I^2^ = 99.14%), highlighting the complexity of generalizing anxiety prevalence across diverse sex worker populations. The model indicated a non-significant pooled effect size (*p* = 0.0686), suggesting that while there might be a trend toward increased anxiety levels, this did not uniformly manifest across all included studies. This result, coupled with the high degree of variability (tau^2^ = 2.4458), signals the multifaceted nature of anxiety prevalence among sex workers, necessitating a closer examination of specific subgroup characteristics that might influence these outcomes. Please refer to Figure 3 for the forest plot showcasing the prevalence of subclinical anxiety or anxiety disorder.

#### 3.11.2 Subgroup analysis: legal status of sex work

The mixed-effects model exploring the impact of legal status on anxiety prevalence revealed that this factor did not significantly explain the heterogeneity observed across studies (*p* = 0.6247).

#### 3.11.3 Subgroup analysis: population

Further subgroup analysis focused on specific demographics within the sex worker population, including those living with HIV, drug-using sex workers, female sex workers, migrant sex workers, and survivors of sex trafficking. This analysis highlighted significant residual heterogeneity (I^2^ = 99.31%), indicating considerable variability in anxiety prevalence across these groups. Subpopulation was not a significant mediator of anxiety prevalence (*p* = 0.0526).

#### 3.11.4 Subgroup analysis: economic conditions

The examination of economic conditions did not yield significant findings (*p* = 0.8794). This analysis suggests that within the context of this meta-analysis, economic conditions do not have a significant direct impact on anxiety prevalence among sex workers.

#### 3.11.5 Subgroup analysis: assessment method

The subgroup analysis revealed that the assessment method is not a significant moderator (*p* = 0.0358) given the adjusted significance level of *α* = 0.0042. The results indicated significant residual heterogeneity (I^2^ = 98.84%).

#### 3.11.6 Assessment of publication Bias

**Figure 4:**
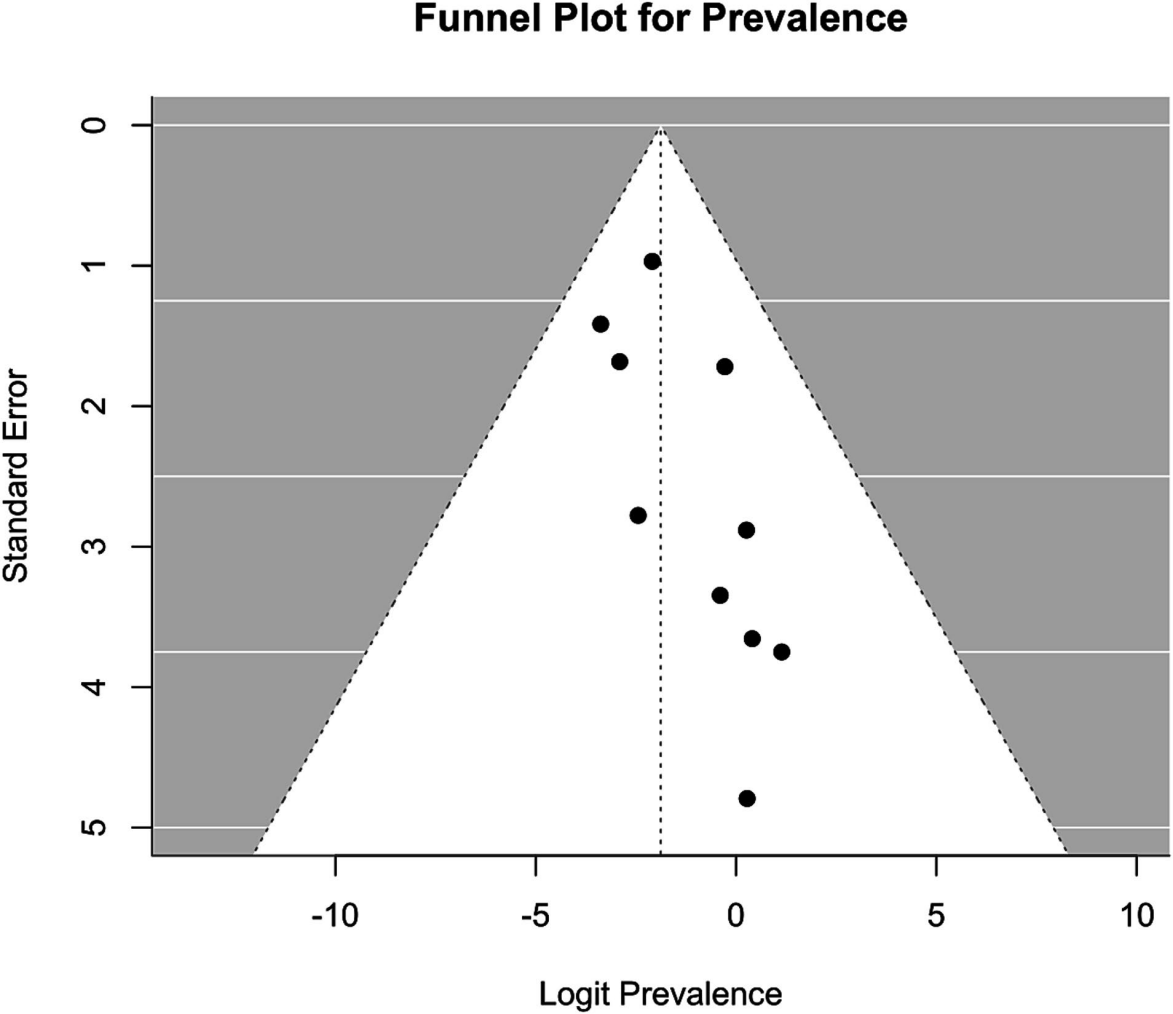
Funnel plot assessing publication bias in logit-transformed subclinical anxiety and anxiety disorder prevalences (X-axis) and standard error (Y-axis).

To evaluate the potential impact of publication bias on our meta-analysis results, we conducted Egger’s test. Upon conducting Egger’s test, we obtained a *p*-value of 0.6297748. This non-significant result indicates no strong evidence of funnel plot asymmetry or publication bias in our meta-analysis dataset. Please refer to Figure 4 for the funnel plot. The absence of significant publication bias provides increased confidence in the robustness of our meta-analysis findings.

### 3.12 Depression

#### 3.12.1 Overall meta-analysis findings

**Figure 5:**
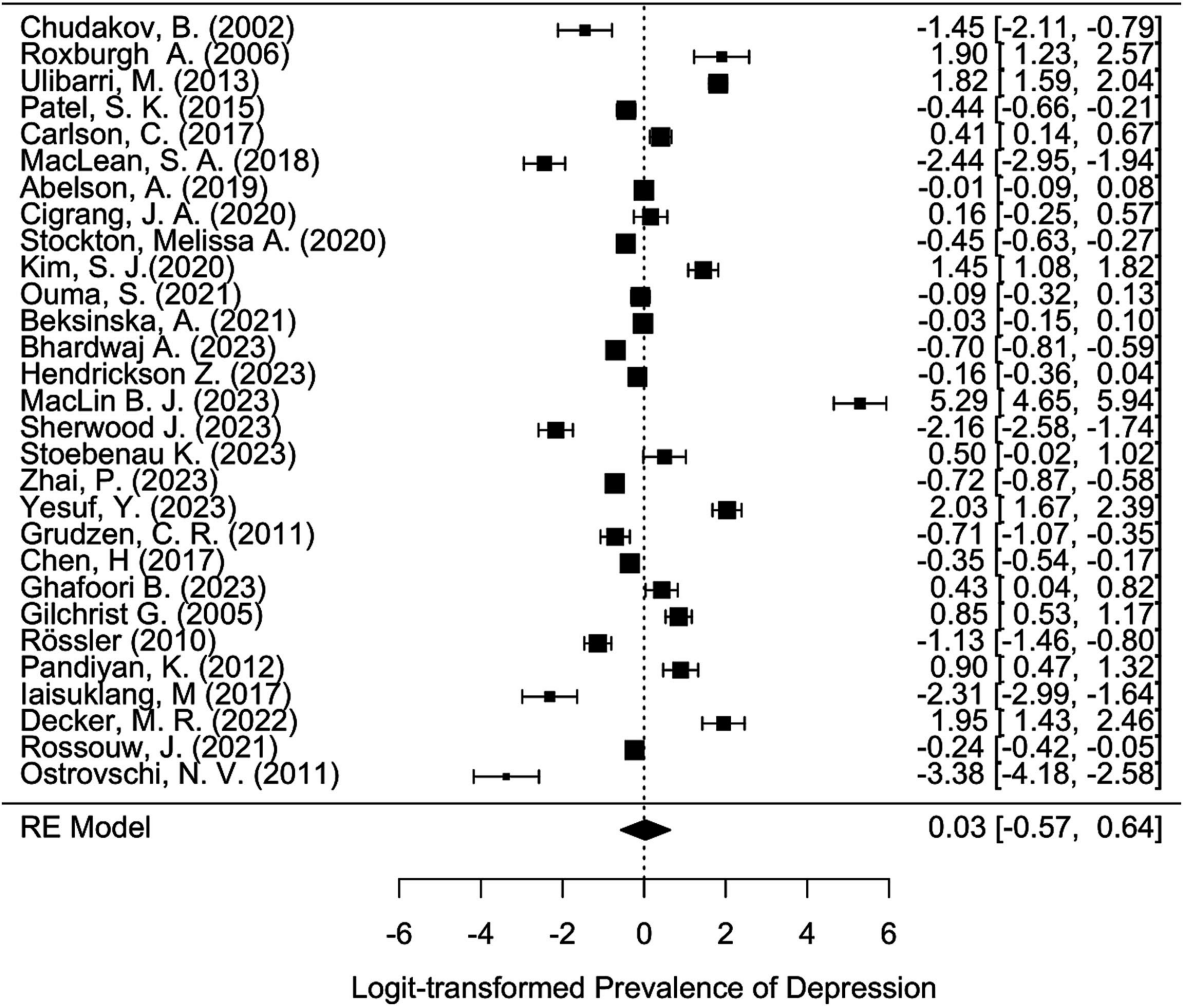
Forest plot showing the logit-transformed prevalence of depression and calculated confidence intervals across the included studies.

The meta-analysis, based on 29 studies, utilized a random-effects model to account for variability across studies, indicating a high degree of heterogeneity (I^2^ = 99.51%, H^2^ = 203.65) in depression prevalence among sex workers. This heterogeneity is further quantified by the significant tau^2^ value (2.7128), suggesting substantial differences in depression rates across different contexts and populations. The overall effect size estimate was not significant (*p* = 0.9141). Please refer to Figure 5 for the forest plot.

#### 3.12.2 Subgroup analysis: legal status of sex work

The analysis of moderators revealed that the legal status of sex work did not significantly affect the prevalence of depression. The test for the overall effect of the moderators [QM (df = 2) = 0.8794, *p* = 0.6442] indicated that differences in legal status do not statistically alter the effect sizes.

#### 3.12.3 Subgroup analysis: population

The mixed-effects model for population subgroups female sex workers, street-based female sex workers, female sex workers who reported injection drug use, female sex workers who were arrested for at least one drug offense, female sex workers living with HIV, survivors of sex trafficking, adult entertainment film performers, and migrant female sex workers highlights significant differences in depression prevalence among distinct groups. Particularly, one subgroup (sex workers with HIV) showed a markedly higher depression prevalence, with an effect size of 5.2933 and a significant *p*-value (<0.0001), suggesting specific population characteristics or conditions that markedly elevate depression risk. Other subgroups did not show significant differences, suggesting that while certain population characteristics can substantially impact depression prevalence, this is not universally applicable across all sex worker populations.

#### 3.12.4 Subgroup analysis: economic conditions

The subgroup analysis based on GDP did not reveal significant differences in depression prevalence among sex workers (*p* = 0.7336).

#### 3.12.5 Subgroup analysis: assessment method

The moderator analysis indicated that conducting a clinical interview was not a significant moderator (*p* = 0.1330). Furthermore, the results showed significant residual heterogeneity (I^2^ = 99.94%).

#### 3.12.6 Assessment of publication Bias

**Figure 6:**
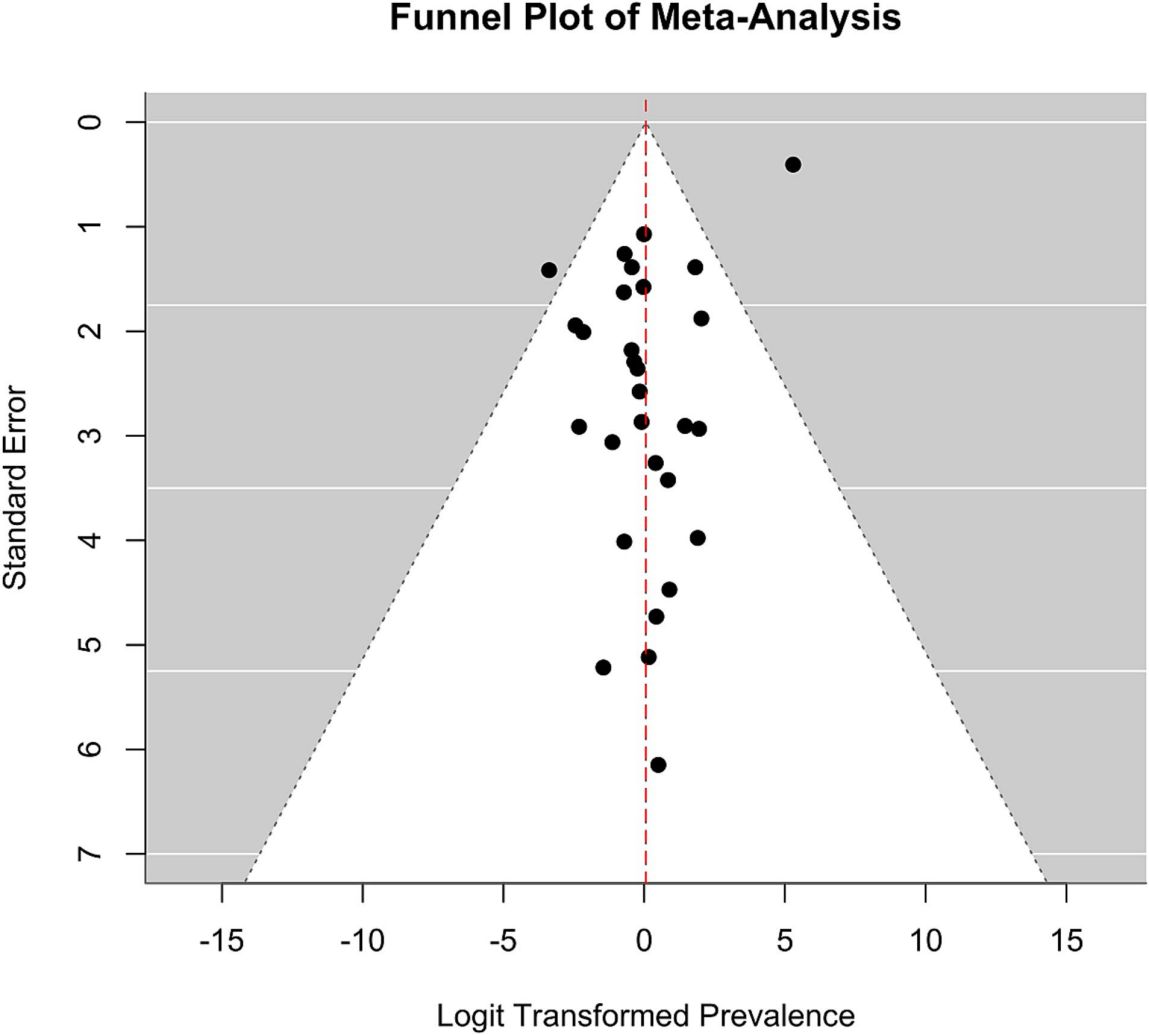
Funnel plot assessing publication bias in logit-transformed depression prevalences (X-axis) and standard error (Y-axis).

A critical examination of publication bias was conducted through Egger’s test. The regression analysis yielded a t-statistic of −3.8726 with 27 degrees of freedom, resulting in a highly significant p-value of 0.0006. This significant result indicates a substantial asymmetry in the funnel plot, suggesting the presence of publication bias. The intercept of the regression, estimated at 94.6129 with a 95% confidence interval ranging from 78.3130 to 110.9129, further substantiates this bias. The substantial deviation of this intercept from zero highlights an overrepresentation of studies with larger, more significant effects. Please refer to Figure 6 for the funnel plot.

### 3.13 PTSD

#### 3.13.1 Overall meta-analysis findings

**Figure 7:**
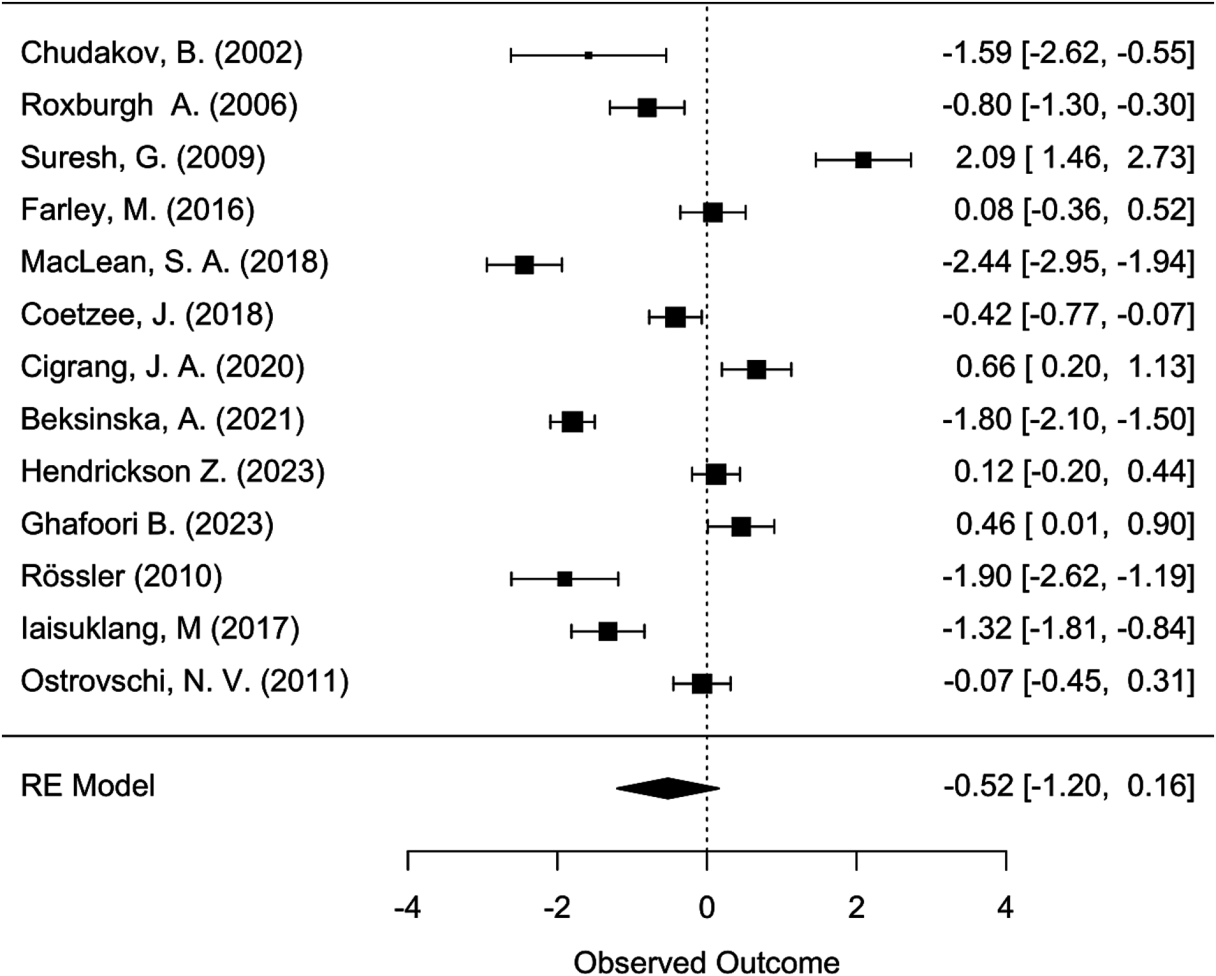
Forest plot showing the logit-transformed prevalence of PTSD and calculated confidence intervals across the included studies.

The primary analysis utilized a random-effects model to account for the inherent heterogeneity across the included studies (k = 13). The findings indicate a high degree of variability (I^2^ = 96.73%), with a tau^2^ estimator of 1.4986, suggesting significant differences in PTSD prevalence among sex workers across different contexts. Despite this variability, the pooled effect size was not statistically significant (*p* = 0.1325). Please refer to Figure 7 for the forest plot.

#### 3.13.2 Subgroup analysis: legal status of sex work

Exploring the influence of the legal status of sex work on PTSD prevalence, the analysis did not reveal significant effects across categories (illegal, legal, and partially legal). This suggests that within the scope of this analysis, the legal framework surrounding sex work does not directly correlate with PTSD prevalence rates among sex workers. The residual heterogeneity (I^2^ = 97.17%) remained high.

#### 3.13.3 Subgroup analysis: sample population

A mixed-effects model was employed to delve into the PTSD prevalence across different sample populations within the sex worker community. The analysis revealed a notably lower PTSD prevalence among female sex workers (*p* < 0.0001) compared to other subgroups (street-based female sex workers, American Indian/Alaska Native women in sex work, female sex workers who were arrested for at least one drug offense and survivors of sex trafficking). The subgroup analysis underscores the substantial heterogeneity and the role of specific population characteristics in shaping PTSD prevalence, with a significant portion of the variability in PTSD rates across studies (I^2^ = 93.3%) remaining unaccounted for by this model alone.

#### 3.13.4 Subgroup analysis: economic conditions

The investigation into the impact of GDP on PTSD prevalence also did not yield significant results (*p* = 0.7639).

#### 3.13.5 Subgroup analysis: assessment method

The analysis showed that conducting a clinical interview as an assessment method for PTSD did not significantly alter the reported prevalence of the condition in the included studies (*p* = 0.8722).

#### 3.13.6 Assessment of publication bias

**Figure 8:**
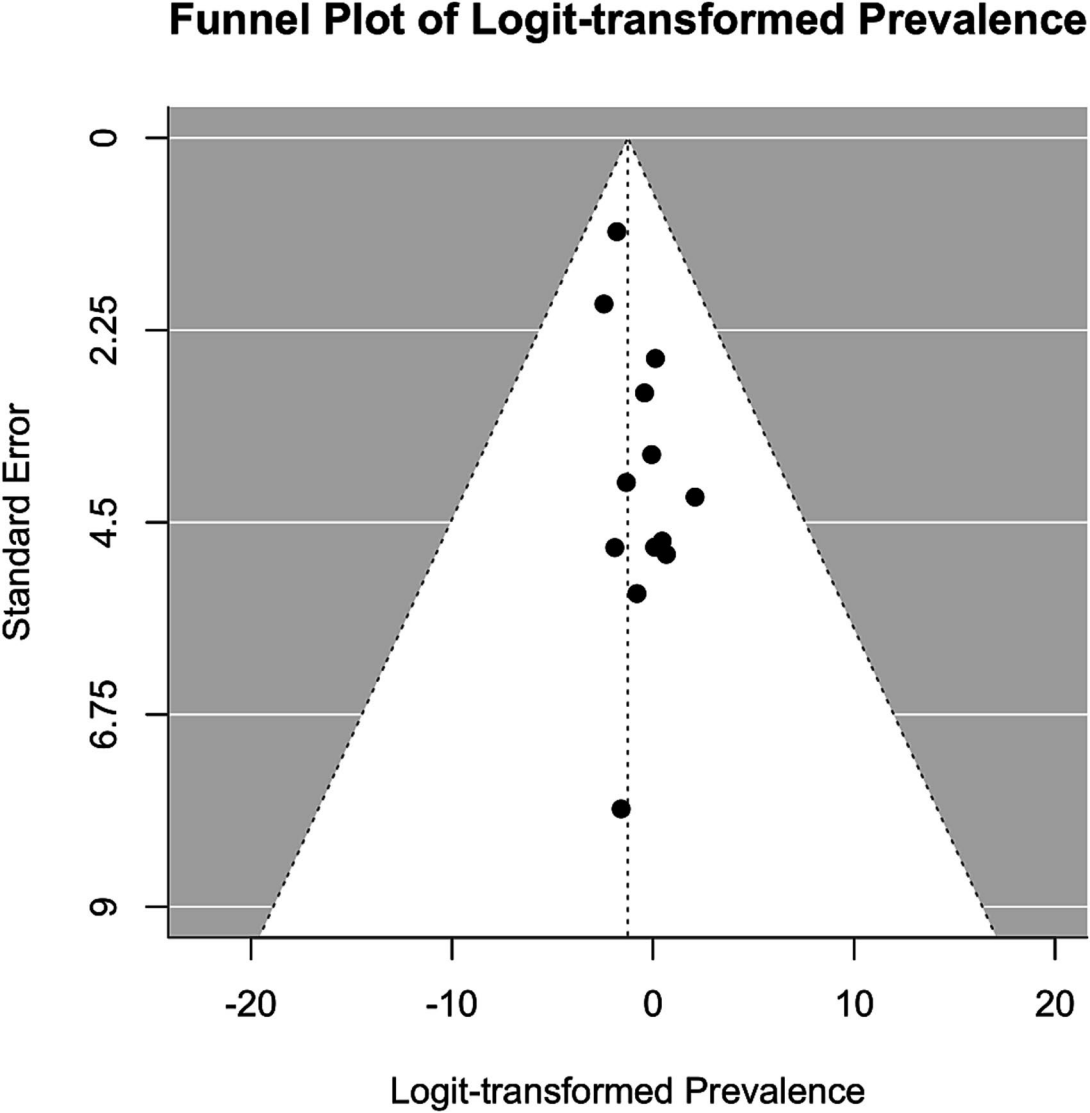
Funnel plot assessing publication bias in logit-transformed PTSD prevalences (X-axis) and standard error (Y-axis).

Our analysis, utilizing Egger’s test, yielded a non-significant *p*-value of 0.0958, along with an estimated asymmetry coefficient of −0.2459 (95% CI: −0.8599, 0.3681). These results do not reach the necessary levels for statistical significance. Please refer to Figure 8 for the funnel plot.

## 4 Discussion

### 4.1 Overall quality of included studies

This comprehensive examination delves into the mental health challenges prevalent among sex workers globally. The overall quality of the included studies, as assessed using the Newcastle-Ottawa Scale (NOS), varied significantly across the review. Some studies demonstrated robust methodological rigor, receiving high scores in categories such as selection of cohorts and ascertainment of exposure or outcome, reflecting their strong design and low risk of bias (111). These studies were characterized by well-defined populations, clear selection criteria, and thorough follow-up procedures (68, 103). However, other studies scored lower due to limitations in areas such as comparability and small sample sizes (9, 40, 44, 85, 88). Despite these differences in quality, the overall body of evidence provides valuable insights, though findings from lower-quality studies should be interpreted with caution, acknowledging their greater risk of bias (see Supplementary Table 3).

### 4.2 High heterogeneity of findings across included studies and potential explanations

Through an exploration of mental health conditions such as anxiety, depression, PTSD, substance use, and dependence, the study reveals significant heterogeneity in prevalence rates. Such diversity underscores the complex mental health landscape faced by sex workers and underscores the multifaceted influences of socio-economic, and individual factors on their wellbeing. While the tables provide the necessary details, the narrative summary may lack depth in terms of contextualization. This limitation should be recognized, as it may hinder the reader’s ability to fully grasp the nuances of each study. Future reviews with a more focused scope or fewer included studies could potentially address this issue more comprehensively.

Legal frameworks and economic conditions did not show any mediating effect on mental health outcomes in the meta-analysis while the sub-population was a significant mediator in the context of PTSD and depression. The findings from the meta-analysis align well with individual studies. It is shown throughout numerous studies that street-based sex workers experience more violence than other sex workers. This aligns well with our meta-analysis finding showing that sub-population is a significant mediator of PTSD prevalence (124, 125). In this study, GDP per capita was used as a proxy for the economic conditions of a country, with the understanding that it broadly correlates with access to essential resources such as food, water, and healthcare, which were shown to be relevant factors influencing the mental health of sex workers (35). Although GDP per capita does not directly measure access to resources, it is commonly used as an indicator of a country’s overall economic capacity to supply them. Future research might benefit from incorporating more direct indicators of resource access to further refine our understanding of these relationships.

Specific demographic segments within the sex worker community, such as those living with HIV and those engaged in substance use, display higher prevalence rates for certain mental health conditions. This observation suggests the necessity for targeted interventions that address the unique vulnerabilities of these subgroups, underscoring the importance of nuanced, population-specific health services. These findings display, that sex workers should not be viewed as a collective when planning policies or interventions. “Sex work” must be understood as a vague umbrella term that comprises different subgroups that have differing needs due to varying experiences and life realities. Policymakers and stakeholders are urged to prioritize local and context-specific mental health services for sex workers.

The study’s findings are subject to several severe limitations, chiefly arising from the inherent heterogeneity among the included researches. This heterogeneity, reflecting differences in methodologies, populations studied, and geographic locations, poses significant challenges to generalizing the results across the global context of sex work. It is especially important to acknowledge the heterogeneity arising from including both subclinical anxiety and anxiety disorders in the meta-analysis. The analysis hence does not purely provide insights on prevalence of anxiety disorder but rather investigates whether sex workers tend to experience increased levels of anxiety. By combining studies with varying definitions and thresholds for anxiety, our analysis introduces a degree of heterogeneity that could obscure important differences between subclinical and clinical levels of anxiety. Despite this, we believe that this inclusive strategy is necessary to generate preliminary insights in an under-researched field, while acknowledging that future studies should aim for more standardized methodologies to enhance the precision and comparability of findings. To further examine the relationship between assessment methods and the observed outcomes, we tested whether clinical interviews, compared to other self-assessment or alternative methods, had a mediating effect.

While is a known phenomenon in psychological or psychiatric research that sel--assessment methods tend to lead to higher outcome variables and effect sizes, clinical interviews did not significantly mediate the prevalence rates in our analysis, suggesting that differences among the differing self-assessment tools or other factors must account for the variability between the included studies.

E.g., a significant source of heterogeneity in the studies analyzed could also be attributed to cultural differences. Cultural norms and values shape societal attitudes toward sex work, the stigma associated with it, and the availability of support systems for sex workers, all of which can profoundly impact mental health outcomes. While it was not feasible to statistically control for country or cultural factors as moderators in this meta-analysis, the role of culture cannot be overlooked. Further research is needed to clarify the impact of cultural aspects on the mental health of sex workers. While it would be valuable to compare the prevalence rates of the mental diseases among sex workers to those of the general public, this study does not include such comparisons due to the vast number of studies analyzed and the challenges in obtaining reliable, corresponding prevalence data from the general population for each specific country and year. The lack of available data for many regions and time periods limits our ability to provide a direct comparison between the mental health outcomes of sex workers and the general public.

The detected publication bias regarding the prevalence of depression is a critical concern as it suggests that the meta-analytic results might be skewed toward more positive or statistically significant outcomes. This skew could potentially overestimate the true effect sizes. The findings necessitate a cautious interpretation of the meta-analysis results, with an understanding that the true effects might be more nuanced than reported. Studies reporting high rates of depression among sex workers may have a higher likelihood of publication due to the perceived social significance of the issue, whereas studies indicating lower prevalence might go unpublished. Additionally, research reporting high prevalence rates may be prioritized for publication to emphasize the unmet sociopolitical needs and health disparities experienced by sex workers. In contrast, studies indicating lower prevalence might be viewed as less politically significant or misaligned with advocacy objectives.

### 4.3 Findings of this systematic review and meta-analysis in comparison to other reviews

Our findings align well with findings from other systematic reviews on the mental health of female sex workers (8). However, by expanding the period for included studies (2002–2023) as well as by conducting a meta-analysis in addition to the review we exceed the prior study scopes and provide new scientific insights. In comparison to Martin-Romo et al.’s (8) work we did not investigate general distress, or quality of life as primary outcomes, in order to maintain a clear focus on mental disease and psychiatric relevance. While risk factors for mental health were reported based on “work-related” or “personal” circumstances in the review named above, we decided to report both types of risk factors according to the related mental conditions. Thereby, we provide a detailed picture of risk and resilience factors for mental disorders among female sex workers tailored primarily to inform clinicians and secondary to inform other stakeholders such as policymakers.

## 5 Conclusion and further research directions

The compelling need for further research exploring the intricate relationships between legal status, socio-economic factors, individual vulnerabilities, and the mental health of sex workers is evident. Future studies should aim to bridge the existing gaps in the literature, especially focusing on under-researched regions (see Figure 2) and marginalized subgroups, to provide a more comprehensive understanding of the mental health landscape among sex workers. Marginalized subgroups comprise, e.g., sex workers with HIV, migrant sex workers, survivors of human trafficking, drug-using sex workers, as well as street-based sex workers. Further research must be conducted to investigate the mental health of male and transgender sex workers, as their experience probably differs in certain aspects from the experience of cis-gender female sex workers.

In conclusion, while this study offers valuable insights into the mental health challenges prevalent among sex workers globally, the limitations outlined above must be considered when interpreting the findings. These limitations underscore the importance of future research that addresses the identified gaps, employs standardized measurement tools, and considers the role of unmeasured confounding factors. Such efforts will enhance our understanding of the mental health landscape of sex workers and inform the development of targeted interventions and policy reforms.

## Data Availability

The original contributions presented in the study are included in the article/Supplementary material, further inquiries can be directed to the corresponding author.
