# Supplementary material for: Prevalence, risk and resilience factors of mental health conditions among female sex workers: a systematic review and meta-analysis
Source: Front Public Health. 2025 Jan 13;12:1455999. doi: 10.3389/fpubh.2024.1455999 (PMC11773154; doi:10.3389/fpubh.2024.1455999)
Supplement: Supplementary file 1 [file Table_1.DOCX]

Search strategy used in the present study:

| Database | Search String |
| --- | --- |
| Web of Science | (depression OR anxiety OR suicidality OR psychosis OR "post-traumatic stress" OR suicid* OR depress* OR anxie* OR "mental health" OR "mental disorder" OR "psychopathology" OR "risk factor" OR "resilience factor" OR resilien* OR “mental health” [mesh] OR "Mental dis"[Mesh] OR "anxiety dis"[Mesh] OR "stress dis post traumatic"[Mesh] OR “psychopathol” [Mesh] OR depression [Mesh] OR anxiety [Mesh] OR “risk factor” [Mesh] OR “psychotic dis” [Mesh]) AND ("sex work" OR "prostitution" OR "street sex" OR "paid sex" OR "sex industry" OR “sex work” [Mesh]) |
| PubPsych | **(depression OR anxiety OR suicidality OR psychosis OR "post-traumatic stress" OR suicid* OR depress* OR anxie* OR "mental health" OR "mental disorder" OR "psychopathology" OR "risk factor" OR "resilience factor" OR resilien* OR “mental health” [mesh] OR "Mental dis"[Mesh] OR "anxiety dis"[Mesh] OR "stress dis post traumatic"[Mesh] OR “psychopathol” [Mesh] OR depression [Mesh] OR anxiety [Mesh] OR “risk factor” [Mesh] OR “psychotic dis” [Mesh]) AND ("sex work" OR "prostitution" OR "street sex" OR "paid sex" OR "sex industry" OR “sex work” [Mesh]) PY>=2002 PY<=2022** |
| Ovid | **1) depression/**  **2) anxiety disorder/ or mental disease/**  **3) posttraumatic stress disorder/ or sexual trauma/**  **4) suicidal behavior/ or behavior disorder/**  **5) psychosis/ or mental disease/**  **6) mental health/ or psychological well-being/**  **7) mental disease/**  **8) mental disorder/**  **9) risk factor/**  **10) psychological resilience scale/**  **11) coping behavior/**  **12) prostitution/**  **13) sex work/**  **14) (1 or 2 or 3 or 4 or 5 or 6 or 7 or 8 or 9 or 10 or 11) and (12 or 13)** |
| PsycInfo via EBSCOhost | **(depression OR anxiety OR suicidality OR psychosis OR "post-traumatic stress" OR suicid* OR depress* OR anxie* OR "mental health" OR "mental disorder" OR "psychopathology" OR "risk factor" OR "resilience factor" OR resilien*) AND ("sex work" OR "prostitution" OR "street sex" OR "paid sex" OR "sex industry")** |
| Cochrane Library | **((depression OR anxiety OR suicidality OR psychosis OR "post-traumatic stress" OR suicid* OR depress* OR anxie* OR "mental health" OR "mental disorder" OR "psychopathology" OR "risk factor" OR "resilience factor" OR resilien*) AND ("sex work" OR "prostitution" OR "street sex" OR "paid sex" OR "sex industry" OR "commercial sex"))"** |
